# Hybrid membrane-coated nanosuspensions for multi-modal anti-glioma therapy via drug and antigen delivery

**DOI:** 10.1186/s12951-021-01110-0

**Published:** 2021-11-20

**Authors:** Wenyan Hao, Yuexin Cui, Yueyue Fan, Mengyu Chen, Guobao Yang, Yuli Wang, Meiyan Yang, Zhiping Li, Wei Gong, Yang Yang, Chunsheng Gao

**Affiliations:** State Key Laboratory of Toxicology and Medical Countermeasures, Beijing Institute of Pharmacology and Toxicology, Beijing, 100850 People’s Republic of China

**Keywords:** Glioma, Blood–brain barrier, Biomimetic nanosuspensions, Chemotherapy, Immunotherapy

## Abstract

**Background:**

Glioma is one of the deadliest human cancers. Although many therapeutic strategies for glioma have been explored, these strategies are seldom used in the clinic. The challenges facing the treatment of glioma not only involve the development of chemotherapeutic drugs and immunotherapeutic agents, but also the lack of a powerful platform that could deliver these two moieties to the targeted sites. Herein, we developed chemoimmunotherapy delivery vehicles based on C6 cell membranes and DC membranes to create hybrid membrane-coated DTX nanosuspensions (DNS-[C6&DC]m).

**Results:**

Results demonstrated successful hybrid membrane fusion and nanosuspension functionalization, and DNS-[C6&DC]m could be used for different modes of anti-glioma therapy. For drug delivery, membrane coating could be applied to target the source cancer cells via a homotypic-targeting mechanism of the C6 cell membrane. For cancer immunotherapy, biomimetic nanosuspension enabled an immune response based on the professional antigen-presenting characteristic of the dendritic cell membrane (DCm), which carry the full array of cancer cell membrane antigens and facilitate the uptake of membrane-bound tumor antigens for efficient presentation and downstream immune n.

**Conclusion:**

DNS-[C6&DC]m is a multifunctional biomimetic nano-drug delivery system with the potential to treat gliomas through tumor-targeted drug delivery combined with immunotherapy, thereby presenting a promising approach that may be utilized for multiple modes of cancer therapy.

**Graphical Abstract:**

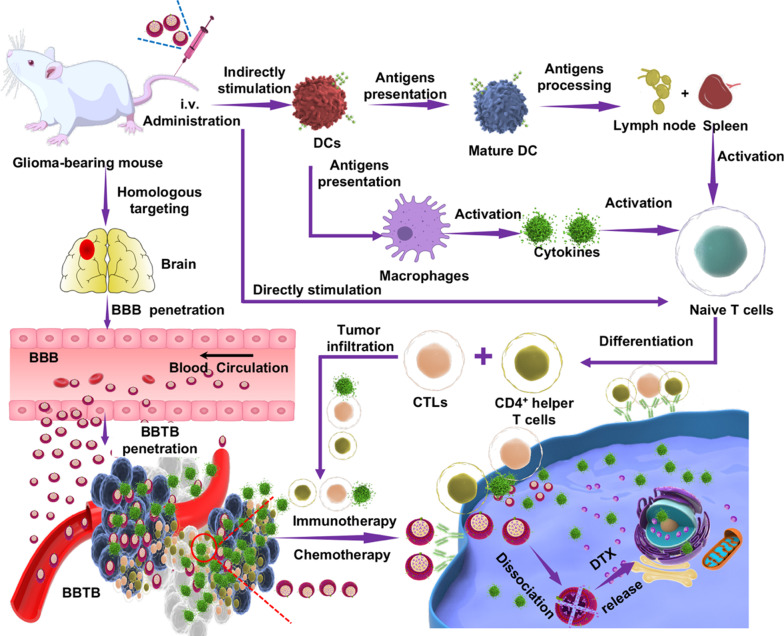

**Supplementary Information:**

The online version contains supplementary material available at 10.1186/s12951-021-01110-0.

## Background

Glioma is one of the deadliest human cancers, with a median survival time of less than 24 months and a 5-year survival rate of only 2–4% [1, 2]. This short survival expectation has drawn significant interest from doctors and scientists developing combinatorial therapies, especially chemotherapy and immunotherapy combination treatment, to improve patient outcomes [3]. While other tumors have responded favorably to trials combining immunotherapy and chemotherapy, glioma remains uniformly deadly with minimal increases in overall survival. Gliomas differ from others because they are isolated behind the physiological barrier (the blood–brain barrier, BBB) and the pathological barrier (blood–brain tumor barrier, BBTB), harbor increased heterogeneity and mutational burden, and cause immunosuppression from the brain environment and tumor itself [4, 5]. Given these limitations, chemotherapy and immune checkpoint blockade agents reach the glioma itself in very limited amounts. Therefore, the challenges facing the treatment of glioma not only involve the development of chemotherapeutic drugs and immunotherapeutic agents, but also the lack of a powerful platform that could deliver these two moieties to the targeted sites.

To date, many nanotechnology-based delivery systems (NDDSs) [6, 7] have been developed for the co-delivery of these two moieties; however, none of these systems have been approved by the US Food and Drug Administration for treating central nervous system (CNS) diseases [8]. Currently, almost all NDDSs essentially depend on the function of the materials they are made of, to target tumors; however, these materials only serve as excipients without any innate therapeutic efficacy. Tailor-made materials or the co-loading of multi-therapeutic agents often involve complex fabrication processes, which may cause variations in batch-to-batch reproducibility and reduce the stability of NDDSs, often resulting in unsatisfactory therapeutic outcomes [9]. Moreover, these systems suffer from the inherent limitations of synthetic materials, such as potential adverse side effects. Therefore, the design of a simple yet effective and safe NDDS is of great significance for clinical translation.

New frontiers in the field of NDDS are advancing research on biomimetic nanocarriers. Among these, cell membrane-coated nanoparticles (NPs) have been widely investigated for cancer therapy, ranging from enhanced efficacy in cancer drug delivery to enhanced immunogenicity of cancer vaccines [10–12]. For drug delivery, these types of biomimetic NPs exhibit unique functions from the source cells, providing an alternative strategy for overcoming biological obstacles and improving drug delivery efficiency [13, 14]. However, for these biomimetic NPs, a large quantity of synthetic materials is required as a scaffold, often resulting in low drug loading yields. High drug loading can help increase the drug concentration at the target site, which helps to achieve an improved tumor inhibition effect. In this study, we extended cell membrane-coating technology to nanosuspensions. Similarly to a “pure drug particle,” nanosuspensions have high drug carrying capacity due to the absence of a carrier, which helps to boost drug concentration at the targeted sites, and are readily translatable to the clinical arena [2, 15].

In addition to drug delivery, cell membrane-bound tumor antigens are also used to train the immune system to recognize and fight cancer, and mimic particulate carriers modified with these surface antigens have been previously prepared to improve vaccine efficacy [16–18]. As professional antigen-presenting cells, dendritic cells (DCs) can mobilize a variety of immune resistance mechanisms [19–21]. Mature DCs stimulated by tumor-associated antigens can promote the proliferation and activation of CD8^+^ and CD4^+^ T lymphocytes. Activated CD8^+^ T-lymphocytes (CTLs) specifically recognize tumor cells and induce tumor apoptosis [22, 23]. Moreover, activated CD4^+^ helper T lymphocytes upregulate the expression of major histocompatibility complex (MHC) molecules on the surface of tumor cells, thereby helping CTLs to recognize tumor cells and further induce cancer cell apoptosis [24]. Recently, DC-based immunotherapy has received widespread attention [25]. DC vaccines have been proven effective and safe in patients with glioblastoma, melanoma, renal cell carcinoma, and prostate and ovarian cancers [26]. Unfortunately, based on basic science and clinical trial data of the past 20 years, the efficacy of these vaccines is still far below expectations (only 10–15%) [27]. Analyzing the reasons for this failure, two main factors were identified: (1) Most of the tumor DC vaccines target a single antigen; however, tumor cells can easily escape immune effects against a single epitope through antigen mutation. (2) Extracted tumor proteins are easily degraded by proteolytic enzymes in the body, which leads to unsustainable immune effects and difficulty in obtaining an ideal therapeutic effect, thus complicating the extraction process. Therefore, efficient antigen loading and improved stability are essential for DC vaccine optimization. Generally, cancer cells bear a broad category of antigens, including tumor-associated and tumor-specific antigens, and high levels of tumor antigens within cancer vaccines are required to reach the threshold for T cell recognition, thereby breaking immunological tolerance [28–30]. To maximize tumor-specific immune response, therefore, the cytomembrane vaccine, which was generated based on the fusion of the cytomembranes between cancer cells and DCs, is an exciting strategy that is readily translatable to the clinical arena [16, 31]. In addition, cancer cell membranes have domains that adhere to homologous cells and homologous binding proteins, which can provide nanomedicines coated with cancer cell membranes with tumor-targeting properties [32–34]. More recently, Xu et al. have reported a study of nanoparticles coated with functional DC membrane for combining photodynamic therapy and immunotherapy to terminate tumor growth. This suggested that the presence of tumor-associated antigens and T cell stimulating factors in DCm cloak [35]. Inspired by these natural properties, in this study, we fused membrane materials derived from glioma cells with DCm to create a hybrid biomimetic coating. Compared to cellular vaccines, the hybrid membrane possesses better biosafety, easier large-scale fabrication, and longer storage properties because of the exclusion of genetic materials. Because they are inherited from two parent cell lines, hybrid membranes are expected to confer not only a continuous source of tumor antigens, which can be presented by the DCm for T cell activation and offer strong immune responses against glioma cells, but also the driving force for drug delivery at glioma sites.

**Fig. 1:**
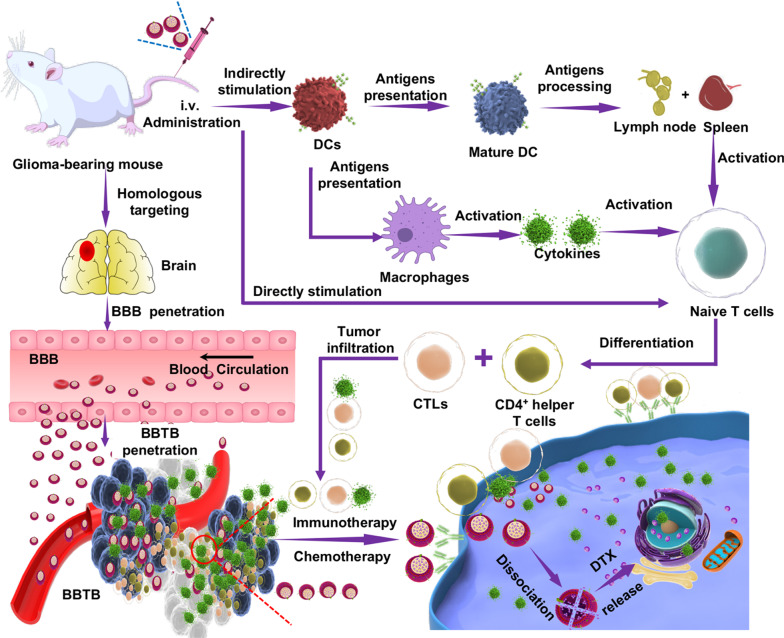
Schematic illustration of the effects of the biomimetic nanosuspensions directly and indirectly, including homologous targeting chemotherapeutics for direct treatment of glioma and enhanced immunotherapy that kills tumors indirectly by activating the body's immune system to maximize the synergistic chemo/immunotherapy

Here, we tested the ability of hybrid membrane-coated nanosuspensions for multiple modes of anti-glioma therapy (Fig. 1). To this end, we developed chemoimmunotherapy delivery vehicles based on C6 cell membranes and DC membranes to create hybrid membrane-coated nanosuspensions (DNS-[C6&DC]m). Specifically, we chose docetaxel (DTX) [36], an insoluble chemotherapeutic drug with severe toxicity, as a model drug for the synthesis of DTX NS (DNS). Although several studies related to biomimetic NPs, including hybrid membrane-coated NPs, have been previously conducted [37–39], most of them focused on monotherapy rather than combinatorial therapies. To our knowledge, this is the first study on the use of hybrid membrane-coated nanosuspensions for multiple modes of anti-glioma therapy via the delivery of high levels of tumor antigens and high doses of drug. The physical and chemical properties of DNS-[C6&DC]m were characterized, and tumor targeting, immune activation ability, glioma inhibitory effect, and biological safety were assessed. Our findings provide valuable preclinical data to validate a noninvasive, efficient biomimetic nanoplatform for chemo-immunotherapy of glioma, an intractable and deadly malignant disease.

## Materials and methods

### Materials

Hypromellose E5 (HPMC E5) and sodium deoxycholate (SDC) were provided by Fenglijingqiu Commerce and Trade Co., Ltd. (Beijing, China). Lecithin was purchased from Yuanye Bio-Technology Co., Ltd. (Shanghai, China). Anti-ICAM, anti-CD44, anti-CD80, anti-MHC I, anti-CD31, anti-CD8a, anti-CD86, and anti-CD4 antibodies were purchased from Abcam (Cambridge, UK). DTX was acquired from Aladdin Biochemical Technology Co., Ltd. (Shanghai, China). All chemical reagents were of analytical grade and were purchased from Macklin Biochemical Co., Ltd.

### Cells and experimental animals

C6 glioma cells, RAW264.7 cells, bEnd.3 cells (mouse brain microvascular endothelial cells), and HUVECs (human umbilical vein endothelial cells) were supplied by the Cell Resource Center of IBMS (Beijing, China) and cultured in Dulbecco’s modified Eagle’s medium (DMEM) containing 10% fetal bovine serum (FBS; Gibco) and 100 IU penicillin.

Both female and male ICR mice (initially weighing 18–22 g) were purchased from SPF Biotechnology Co., Ltd. (permit number: SCXK 2019–0010, Beijing, China).

### Preparation and characterization of DNS

DNS was prepared using an ultrasonic precipitation method [40]. DTX (10 mg) was dissolved in 1 mL ethanol and mixed with 50 mg lecithin as the oil phase. A mixed solution of 0.5 mg/mL HPMC E5 and 4 mg/mL surfactant SDC was used as the water phase. The water phase was then placed in an ice bath ultrasound (800 W). The oil phase was slowly dripped into the water phase, stirred while dripping, and ultrasonicated for 5 min after the dripping was concluded. The organic solvent was volatilized by magnetic stirring in an ice bath for 12 h. The prepared DNS was stored at 4 °C until further use.

The nanoparticle size (diameter, nm) and surface charge (zeta potential, mV) of the prepared formulations were measured using dynamic light scattering (DLS) (Litesizer 500, Anton Parr, Austria). The prepared DNS was stored in 1× phosphate-buffered saline (PBS) or PBS containing 10% FBS at 37 °C to observe its stability, which was analyzed by measuring the particle size and zeta potential within 72 h. The morphology of the DNS was characterized using transmission electron microscopy (TEM; H-7650, Hitachi, Japan). Drug powder was prepared using a freeze-drying method. Briefly, 20 mL of DNS was placed in a 50 mL beaker and pre-frozen at − 80 °C for 4 h, after which it was freeze-dried for 24 h to obtain a solid DNS. DTX, HPMC E5, SDC, lecithin, physical mixture, and solid DNS were acquired for X-ray diffraction (XRD, D8 advance, Bruker, Germany) analysis at 2 °C /min. Scanning angles between 5° and 90° were used to observe the crystal changes of the drug. Further, differential scanning calorimetry was utilized to validate the physical state and crystal shape of the drug, at 10 °C /min, within the temperature range 40–300 °C. Fourier transform infrared absorption spectrometry (FTIR) was used for drug analysis and identification (wave number was 4000–450 cm^−1^) to determine whether the drug molecules were changed.

### Generation of DCs

DCs were isolated from ICR mice. Briefly, bone marrow mesenchymal stem cells were extracted from the bone marrow cavity of mouse femur and tibia bones, and cultured in RPMI-1640 medium containing 20% FBS. The medium contained recombinant IL-4 (10 ng/mL) and GM-CSF (10 ng/mL). After 7 days, DCs were collected for further use.

### Isolation of DC membrane and C6 cancer cell membrane

According to a previously reported method [41], C6 glioma cells were cultured in DMEM medium containing 10% FBS (37 °C, 5% CO_2_). When cell density reached 80–90%, cells were digested with trypsin, centrifuged at 2000 rpm/min to collect cells, and then washed with PBS solution. Tumor cells were dispersed in 25% PBS hypo-osmotic solution containing protease inhibitors, centrifuged at 20,000 rpm to remove cancer cell nuclei and other substances, and then centrifuged again to obtain cancer cell membranes (10,000 rpm). DCs were collected and washed three times with cooled PBS (pH 7.4). Cell pellets were then suspended in a hypotonic lysis buffer containing phenylmethanesulfonyl fluoride and incubated in an ice bath for 15 min according to the manufacturer’s instructions. Subsequently, cells were broken using a repeated freeze–thaw method three times and further centrifuged at 700×*g* for 10 min at 4 °C. The resultant supernatant was further centrifuged at 20,000×*g* for 30 min to collect cracked cell membranes. These cell membrane products were then lyophilized and stored at − 80 °C. The frozen membrane materials were rehydrated in ultrapure water prior to future use. The protein content in the membranes were determined using the bicinchoninic acid (BCA) protein assay to pre- pare DNS-[C6&DC]m.

### Membrane fusion study

To prepare a biomimetic nanosuspension with a hybrid cell membrane as a carrier, first we verified whether it is possible to combine C6m and DCm. To conduct the Förster resonance energy transfer (FRET) study, two lipophilic dyes were employed: 1,1′-dioctadecyl-3,3,3′,3′-tetramethylindodicarbocyanine, 4-chlorobenzenesulfonate salt (DiD, excitation/emission = 644/663 nm) and 1,1′-dioctadecyl-3,3,3′,3′-tetramethylindocarbocyanine perchlorate (DiI, excitation/emission = 549/565 nm). The ultrasound fusion method was utilized for fusing the C6 and DC membranes. C6 cell membrane was stained with DiD and DiI, and with respect to C6 membrane protein, the final ratios were 0.2 and 1.26 wt% for DiI and DiD, respectively. A solution containing the C6 membrane was then added to the vial and stirred at 37 °C for 1 h. Afterward, free dye was washed away by centrifuging the membrane at 10,000×*g* for 15 min, three times. DC membrane was added to the DiI/DiD-doped C6 membrane at DC membrane to C6 membrane protein weight ratios of 5:1, 3:1, 1:1, and 0:1, followed by sonication (300 W, 5 min) to complete membrane fusion. The fluorescence spectrum of each sample was read using a plate reader (Tecan Spark, Austria) with an excitation wavelength of 500–650 nm under different mixing ratios (DCm/C6m). The fluorescence recovery of the donor (C6-DiI) at a low emission peak (nm) was utilized to indicate the increased amount of fusion.

### Preparation and Characterization of DNS-[C6&DC]m

DNS solution (1 mL, 1 mg/mL) was added to [C6&DC]m solution (0.5 mL, 0.3 mg/mL) and sonicated (300 W) for 10 min in an ice bath to achieve the [C6&DC]m coating. The mixture solution was centrifuged at 10,000 rpm for 5 min to remove the excess membrane, and the resulting DNS-[C6&DC]m was resuspended in deionized water for future use. DNS-[C6&DC]m was acquired by ultrasonic treatment for 5 min. The morphology was characterized using TEM, and the size and zeta potential of DNS-[C6&DC]m were measured using DLS. DNS-[C6&DC]m was stored in either 1 × PBS or PBS containing 10% FBS at 37 °C. Stability analysis was carried out by measuring the particle size and zeta potential within 72 h. The drug loading efficiency (LE) and encapsulation efficiency (EE) of DNS-[C6&DC]m were determined by HPLC (Agilent1200, USA) [42, 43]. Briefly, 5 mg (W) of the lyophilized DNS-[C6&DC]m powder was taken into a measuring flask, dissolved in acetonitrile and constant volume to 100 mL, centrifuged at 12,000 r·min-1 for 20 min, 200 μL of supernatant was taken, and the concentration of non-loaded DTX in supernatant was measured by HPLC at 272 nm and calculated based on a pre-established DTX standard curve. LE and EE of DNS-[C6&DC]m can be determined as follows: LE = M_DTX-loaded_/(M_[C6&DC]m_ + M_DTX-loaded_), EE = M_DTX-loaded_/M_DTX-initial_. M_DTX-initial_ is the initial mass of DTX used for DNS-[C6&DC]m preparation. M_[C6&DC]m_ is the mass of [C6&DC]m used for DNS-[C6&DC]m preparation. M_DTX-loaded_ is the mass of DTX loaded in [C6&DC]m, which was determined by subtracting the amount of DTX in the supernatant from M_DTX-initial_.

### Protein determination

SDS–polyacrylamide gel electrophoresis (SDS-PAGE) was employed to examine the protein profile of DNS, DNS-C6m, DNS-DCm, and DNS-[C6&DC]m [32]. In brief, proteins were extracted with RIPA lysis buffer, and the total protein amount was quantified using a BCA protein assay kit. The extracted proteins were then mixed with SDS loading buffer and heated at 100 °C for 10 min. SDS-PAGE buffer was used as the run buffer. An equal amount of protein was added to the well containing 10% SDS-PAGE gel, according to the manufacturer's instructions. For the assembly image, the protein was stained with Coomassie blue and imaged 12 h after water decolorization. Proteins were transferred to polyvinylidene fluoride membranes for western blot analysis. Different samples were treated with anti-CD44, anti-ICAM, anti-MHC I, and anti- CD80, and then anti-mouse IgG secondary antibody conjugated with horseradish peroxidase was further incubated at 37 °C for 1 h. Finally, the immunoreactive proteins were visualized on the film.

### In vitro DTX release

Different DTX formulations (1 mL) loaded in dialysis bags (MW, 8000) were immersed in 50 mL of PBS (pH 7.4), PBS (pH 6.8) or 10% FBS with 0.5% Tween-80 (v/v), respectively. Next, the drug release system was shaken in water bath at 37 °C for 72 h under horizontal shaking at 100 rpm. 100 μL dialysis solution outside of the dialysis bag was take out at different time, and 100 μL of fresh drug-free medium was added into the dialysis solution outside of the dialysis bag. Finally, the content of DTX in solution was detected by HPLC, and the mobile phase was composed of 45% ammonium acetate solution (0.043 mol/L) and 55% acetonitrile. The column was eluted at a flow rate of 1 mL/min at 25 °C [44].

### Cellular uptake

Cellular uptake of different biomimetic nanosuspensions was evaluated in bEnd.3 cells, HUVECs, and C6 glioma cells, and the cells were seeded in a 6-well culture plate at a density of 0.5 × 105 cells/well. After incubation at 37 °C for 24 h, different cell membrane-coated biomimetic nanosuspensions were stained with DiI and incubated with the above-listed cells at a concentration of 2 μg/mL for 15 min, after which they were counter-stained with 4′,6-diamidino-2-phenylinodole (DAPI, Beyotime) for 5 min. After incubation, the cells were fixed with a 4% paraformaldehyde solution. Finally, they were fixed with 50% glycerin. Cell uptake was observed using confocal laser scanning microscopy (CLSM; LSM 880, Zeiss, Germany). For quantitative analysis, 2 × 10^4^ cells were seeded in a 6-well culture plate and cultured for 24 h. After incubation with DiI-labeled biomimetic nanosuspensions for 15 min, the cells were washed three times with cold PBS, trypsinized, resuspended in 300 μL of PBS, and analyzed by flow cytometry (FACSAria III, BD, USA).

### Homotypic targeting

Homologous targeting was verified using the uptake of biomimetic nanosuspensions via different types of cancer cells. Mouse breast cancer cells (4T1), mouse melanoma cells (B16), human hepatoma cells (HepG2), and C6 glioma cells were seeded in confocal laser dishes at a density of 5 × 10^3^ cells/well. DiI-DNS-C6 DC]m was then added to the cell culture medium for 0.5 h. The uptake of different cells was observed using CLSM.

### In vitro penetration of BBB and BBTB

An in vitro BBB model was constructed with bEnd.3 cells using a Transwell cell culture system. Briefly, bEnd.3 cells were seeded in the upper chambers of Transwell cell culture plates (Corning, NY, USA) at 1 × 10^5^ cells/well. The integrity of the cultured monolayer model was tested by measuring the trans-endothelial electrical resistance (> 200 Ω·cm^2^) using a Millicell-ERS voltohmmeter (Millipore). After the cells were cultured to 100% confluence, the transendothelial electrical resistance (TEER) of the cell membrane was recorded. When the TEER was over 200 Ω.cm^2^, C6 cells were seeded in the lower chambers at a density of 1 × 10^5^ cells/well. Next, free DiI, DiI-DNS-DCm, DiI-DNS-C6m, and DNS-[C6&DC]m were added to the upper chamber and cultured for 4 h. Solutions collected from the bottom chamber were assessed using a plate reader, while cell uptake was observed using CLSM. Similarly, HUVECs and C6 cells were used to establish a BBTB model to investigate the penetration ability of the preparations.

The inhibitory effect of DTX on glioma cells in the BBB and BBTB models in vitro was similarly measured using Transwell chambers [45]. In brief, 1.0 × 10^5^ bEnd.3 cells or HUVECs were seeded onto the upper Transwell chamber. When the cells were overgrown, and the TEER was over 200 Ω.cm^2^, the lower chamber was seeded with 2 × 10^3^ C6 glioma cells. Next, free DTX, DNS, DNS-C6m, DNS-DCm, and DNS-[C6&DC]m at DTX concentration of 50 µg/mL, were added to the upper chamber and cultured for 48 h. The inhibition rate of C6 glioma cell proliferation in the bottom chamber was quantified using the Cell Counting Kit-8 (CCK-8) method.

### Immune responses in vitro

To assess the maturation levels of DCs after different treatments, C6m, DCm and[C6&DC]m (50 µg/mL) were incubated with DCs for 48 h. The cells were washed three times with PBS and subsequently stained with anti-CD80-PE, and anti-CD86-APC antibodies (Abcam) for 30 min at 4 °C. After being washed with cold PBS, the cellular fluorescence was detected by flow cytometry. All groups were analyzed in triplicate. Furthermore, to assess the activation levels of T lymphocytes, T cells were treated as above mentioned. After cocultured for 48 h, the T lymphocytes were washed three times with PBS and subsequently stained with anti-CD4-FITC and anti-CD8-PE antibodies (Abcam) for 30 min at 4 °C. After being washed with cold PBS for thrice, the cellular fluorescence was detected by flow cytometry.

To further evaluate whether the treatment in this study can induce immune response from macrophages, C6 glioma cells were seeded in the lower layer of the Transwell chamber, and RAW264.7 cells were seeded in the upper layer of the chamber at a density of 2 × 10^5^ cells/well. After 12 h, C6m, DCm, and [C6&DC]m (50 μg/mL) were added to the upper layer of the chamber. In addition, LPS-treated cells were used as a positive control, while blank medium-treated cells were used as the model group. The suspensions of RAW264.7 cells culture media after stimulation were collected after a 48-h incubation period. The collected samples were tested with mouse IL-6, IFN-γ, and TNF-α ELISA kits, according to the manufacturer’s instructions. The inhibitory effect of cytokines on C6 glioma cells was quantified using the CCK-8 assay.

### In vitro cytotoxicity of DNS-[C6&DC]m on C6 glioma cells

The antitumor activities of DNS, DNS-C6m, DNS-DCm, and DNS-[C6&DC]m were measured using a standard CCK-8 assay. C6 glioma cells were seeded in a 96-well plate at a density of 5 × 10^3^ cells/well. After incubation for 24 h, different DTX formulations (DNS, DNS-C6m, DNS-DCm, and DNS-[C6&DC]m) and free PTX were added at concentrations ranging from 1 to 1,000 μg/mL in 100 μL of medium, and the plates were incubated at 37 °C in a 5% CO_2_ atmospheric condition for 48 h. After incubation for 48 h, 20 μL of CCK-8 solution was added to each well, and the cells were cultured for 2 h. The absorbance of each well was measured at 450 nm using a microplate reader.

### Cell apoptosis assay

Annexin V-FITC/PI dual staining was used to analyze apoptosis. C6 glioma cells were seeded in a 6-well plate at 1 × 10^5^ cells/well and cultured for 12 h. The cells were then treated with different DTX-loaded formulations (equal to DTX 5 μg/mL) or free drugs for 48 h. Following 48 h of incubation, the cells were collected, washed three times with cold PBS, suspended in 300 μL of binding buffer, and stained with Annexin V-FITC/PI. Finally, the cells were analyzed using flow cytometry (FACS Aria III, BD, USA). Non-treated cells were used as negative controls, and the experiment was repeated three times.

### In vivo brain targeting

An intracranial glioma-bearing mouse model was established according to previously described procedures [46]. After anesthetizing C6 cells with chloral hydrate (4%, w/v%), 2 μL of the cells at a density of 2 × 10^6^ were injected into the right striatum (1.8 mm lateral, 1 mm longitudinal, and 4 mm depth) of ICR mice. Using 1,1′-dioctadecyl- 3,3,3′,3′-tetramethylindotricarbocyanine iodide (DiR; 1 mg/kg) as the fluorescent probe, the mice were intravenously injected with free DiR, DiR-DNS-C6m, DiR-DNS-DCm, or DiR-DNS-[C6&DC]m at day 15 post-inoculation (n = 3). At 2, 12, and 24 h, the biodistribution of free DiR and DiR-labeled DTX formulations fluorescence in each group was monitored by an in vivo spectrum imaging system (IVIS® Spectrum, PerkinElmer, USA) using excitation and emission wavelengths of 748 and 780 nm, respectively. For in vitro fluorescence imaging, mice in the DiR-labeled DTX formulations group were sacrificed at 12 h, and their brain tissues and major organs (heart, liver, spleen, lung, and kidney) were collected and visualized using similar imaging parameters. The fluorescence intensity of DiR at each time point was quantitatively evaluated using the ImageJ software.

For tumor distribution analysis, using DiI (0.5 mg/kg) as the fluorescent probe, the glioma-bearing mice were intravenously injected with free DiI, DiI-DNS-C6m, DiI-DNS-DCm, and DiI-DNS-[C6&DC]m. After treatment for 4 h, brain tissues were removed and fixed with 4% paraformaldehyde in the dark for 24 h. DAPI, which was observed at an excitation wavelength of 358 nm and emission wavelength of 461 nm, was used to stain the nucleus. Finally, the distribution of nanosuspensions in each brain tissue was observed using an inverted fluorescence microscope.

### In vivo immune activation

To evaluate the immune response in vivo, the glioma-bearing ICR mice were divided into six experimental groups and immunized three times at intervals of 1 week by intravenous injection with PBS (Model), DNS, DNS-C6m, DNS-DCm, [C6&DC]m, and DNS-[C6&DC]m at an equal amount of DTX (20 mg/kg). After 14 days, the posterior ocular venous plexus blood, which was isolated from mice, was centrifuged (4000 rpm; 10 min) to obtain serum. The different cytokines (TNF-α, IFN-γ, and IL-6) in serum were then quantitatively analyzed. Briefly, serum was diluted three times with the standard diluent. Cytokines were detected using an ELISA kit according to the manufacturer’s protocol. Thereafter, the spleen and draining lymph nodes were dissected and collected. The tissue was fixed with 4% paraformaldehyde, embedded in paraffin, and cut into thin sections. Immunofluorescence and immunohistochemical (IHC) staining were conducted on both lymphoid and spleen tissues to determine the levels of CD4 and CD8 receptors and TNF-α, as well as to observe the activation of the immune system of glioma-bearing mice under different treatments.

### In vivo anti-glioma efficacy

C6 glioma-bearing ICR mice were randomly divided into six treatment groups (six mice/group): PBS, DTX, DNS, DNS-C6m, DNS-DCm, and DNS-[C6&DC]m. The mice were intravenously injected with the corresponding DTX formulations at a DTX dosage of 20 mg/kg every 2 days (Fig. 8A). After administration, brain glioma was evaluated via magnetic resonance imaging (MRI) (PharmaScan 70 T/16, Bruke, US), and the body weight of mice was measured every 2 days after treatment. Mice survival period was also recorded when death or pathologic events occurred. After 20 days of administration, three mice from each group were randomly selected and euthanized, and the brains were collected and fixed at 48 h with 4% paraformaldehyde for H&E, TUNEL, and Caspase-3 staining to determine the degree of tumor cell apoptosis. Brain tissue was stained with IHC to determine the expression of CD31 receptors in neovascularization, which is related to tumor cell proliferation.

### Preliminary safety evaluation in vivo and in vitro

We investigated the in vivo biological safety of biomimetic nanosuspensions. Normal male mice were randomly divided into six groups and intravenously injected with 200 μL of normal PBS, free DTX, DNS, DNS-C6m, DNS-DCm, and DNS-[C6&DC]m. Fifteen days later, all mice were euthanized, and blood cells and serum biochemical indexes were examined. Further, the heart, liver, spleen, lung, kidney, and brain were removed for histology via H&E staining.

### Statistical analysis

All experiments were conducted in triplicate and expressed as mean ± standard deviation. Statistical significance was analyzed using SPSS 19.0 software (IBM Corp., Armonk, NY, USA). One-way analysis of variance with a post-hoc Tukey’s test was used to determine significant differences between datasets. Statistical significance levels were set at *p < 0.05, ** p < 0.01, and *** p < 0.001, ns, not significant, as indicated in Additional file 1: Fig. S1.

## Results and discussion

### Characterization of DNS

**Fig. 2:**
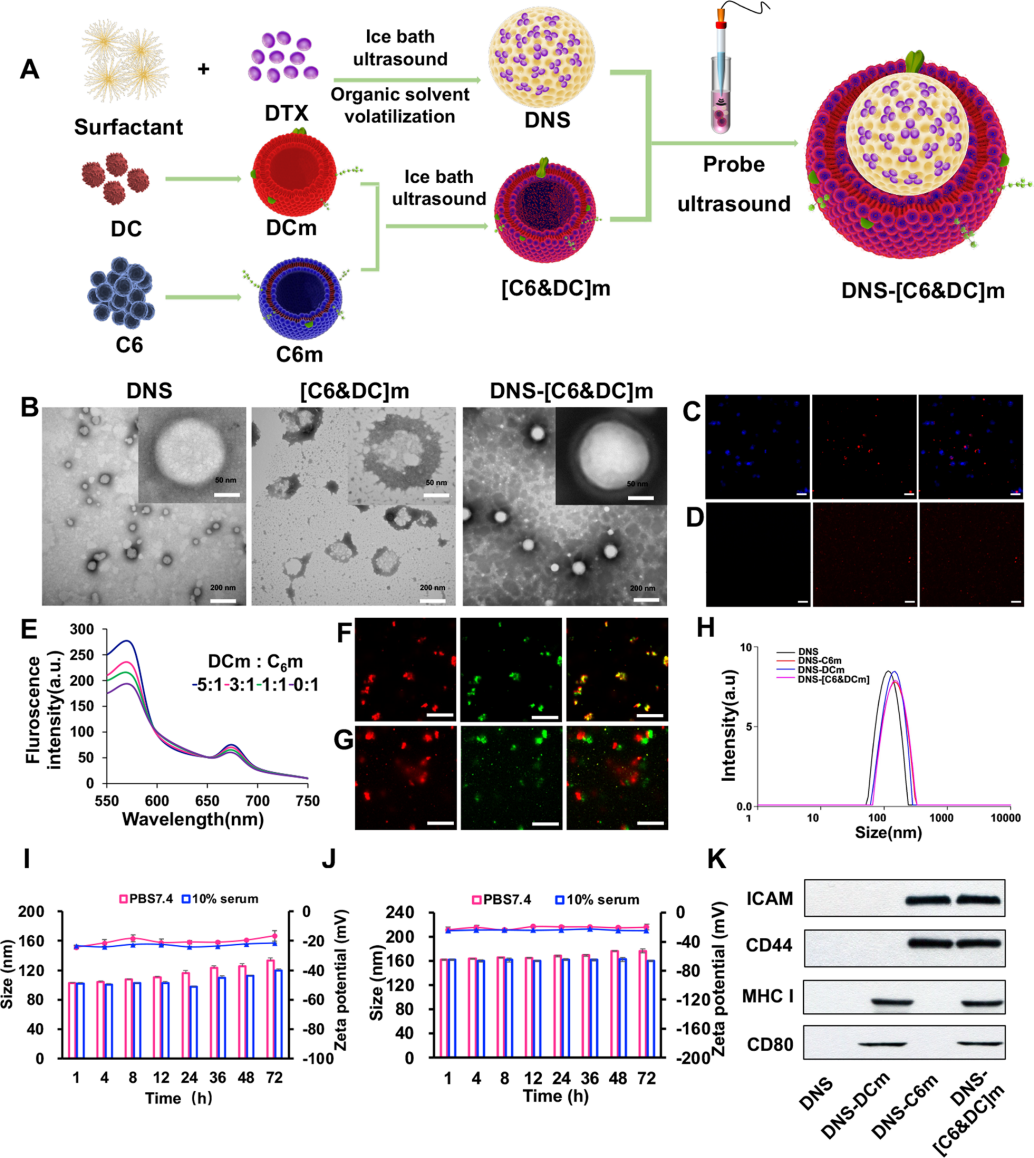
Preparation and characterization of the biomimetic nanosuspensions. **A** Detailed procedure for the preparation of DNS-[C6&DC]m. The extracted C6 glioma cancer cell membrane was hybridized with the DC membrane as the shell structure and then coated onto DNS cores using an ultrasonic fusion method. **B** Morphological appearance of DNS, [C6&DC]m and DNS-[C6&DC]m based on TEM. **C** C6 glioma cell nuclei obtained by low-speed centrifugation in the cell membrane preparation. **D** Cell membranes after purification under high speed centrifugation. Hoechst 33,258 (blue), DiI (red), (60 × magnification). **E** C6 cancer cell membrane was doped with a FRET pair of fluorescent probes and mixed with increasing numbers of DCm. **F** Confocal fluorescent microscopy images of the DNS-[C6&DC]m or either a mixture of DNS-DCm and DNS-C6m (**G**) (red = DCm, green = C6m; scale bar = 200 nm). **H** Particle size distribution of different preparations, as measured using the DLS (n = 3; mean ± SD). **I** Stability of DNS and DNS-[C6&DC]m **J** in pH 7.4 PBS and 10% serum-rich media. **K** Western blot analysis of the expression of membrane-specific proteins, including ICAM, CD44, MHC I, and CD80. All samples were run at equivalent protein concentrations

The process of preparing DNS is illustrated in Fig. 2A. The ultrasonic precipitation method was simple and reproducible. Ethanol can be removed via magnetic stirring, which helps to improve the biological safety of the preparation, and the occurrence of any adverse reactions can be reduced. TEM images demonstrated that DNS was uniform in size and round in shape (Fig. 2B). As shown by the XRD curves (Additional file 1: Fig. S1A), DTX and SDC demonstrate strong crystal diffraction of HPMC E5, and lecithin does not have a diffraction peak, indicating that DTX and SDC exist within the crystal structure. However, HPMC E5 and lecithin are amorphous. The physical mixture showed the same crystal diffraction peak, indicating that simple physical mixing cannot destroy the crystal structures of DTX and SDC. Furthermore, the crystal diffraction peaks in the nanosuspension completely disappeared, showing that DTX exists in an amorphous form in DNS. The same sample was analyzed using differential scanning calorimetry (Additional file 1: Fig. S1B). We discovered that the DTX endothermic peak disappeared in DNS, while the SDC endothermic peak moved forward. DTX is an amorphous structure in DNS, which is consistent with our X-ray diffraction results. The results of FTIR scanning (Additional file 1: Fig. S1C) validated that the drug did not react with excipients in the solution and existed independently. These characterization results validate the successful preparation of the DTX nanosuspension.

### Membrane fusion study

To verify that the extracted C6 cell membrane did not contain tumor nucleus-related genetic material, laser confocal microscopy was used to observe the separation of the nuclear membrane from C6 glioma cells. The nucleus was collected via centrifugation, and a portion of the cell membrane was precipitated. The cell nucleus was removed, the cell membrane was collected, and the nuclear membrane was separated (Fig. 2C, D).

As the successful fusion of DCm and C6m was crucial for the drug and antigen delivery of DNS-[C6&DCm], cell fusion was first investigated. To test the fusion of the DC and C6 membranes, a C6 membrane was doped with two different dyes that constituted a FRET pair [47]. It was observed that the fluorescence resonance occurred at 578 nm, and as the amount of DC membrane increased, there was a recovery of fluorescence at approximately a 675-nm emission wavelength, indicating the fusion of the two membrane materials, weakening FRET interactions in the original C6 membrane (Fig. 2E). Next, laser confocal colocalization imaging was used to further verify the fusion of the two cell membranes. C6m and DCm were mixed in a protein weight ratio of 1:1. The DCm labeled with the red fluorescent dye (DiI) was fused with C6m, which was labeled with a green fluorescent dye (DiD). When a diluted solution of DNS-[C6&DC]m was viewed under confocal microscope, significant colocalization of fluorescent signals was observed (Fig. 2F). In contrast, a mixture of DNS-C6m and DNS-DCm fabricated with individual fluorescently labeled membranes exhibited distinct red and green punctates. It was further demonstrated that C6m and DCm were retained on the DNS-[C6&DC]m at a ratio nearly identical to the 1:1 input (Fig. 2G). These results indicate that it was indeed possible to fuse the two types of cell membranes and incorporate materials of both membranes into the same nanosuspensions.

### Preparation and characterization of DNS-[C6&DC]m

TEM imaging showed that DNS-[C6&DC]m were spherical. A layer of membrane structure was observed on the surface of the nanoparticles, which had a core–shell structure (Fig. 2B). Particle size is an important parameter associated with nanosuspensions, which helps to determine the physical and chemical properties of each particle [48]. DLS was used to compare the sizes of the DNS and DNS-[C6&DC]m. As shown in Fig. 2H, DNS exhibited an original size of approximately 144 nm, which increased by 10 nm after coating [C6&DC]m. This particle size is suitable for molecules in the blood to enter tissue, exist close to cell surface receptors, and promote intracellular transport [49, 50], as observed in our TEM detection results. Additionally, the zeta potential changed from − 20 to − 33 mV (Additional file 1: Fig. S2), which was more conducive to maintaining the stability of the nanosystem. Moreover, the increase in zeta potential can help promote tumor penetration and lysosome escape, which enhances the delivery of brain-targeted drugs. Since stability is a prerequisite for further application of DNS-[C6&DC]m in vivo*,* the agglomerations of the biomimetic nanosystems in PBS and PBS with 10% FBS were evaluated within 72 h at 37 °C to mimic the stability at in vivo conditions. Compared with DNS, DNS-[C6&DC]m maintained good dimensional stability and a negative zeta potential in neutral PBS or PBS containing 10% FBS (Fig. 2I, J), implying that DNS-[C6&DC]m is able to prolong the blood circulation time. These results suggest that the hybrid membrane can significantly improve the stability of DNS and prolong circulation times, which are attributed to a shielding effect that is based on cell membrane coverage. The drug loading efficiency (LE) and encapsulation efficiency (EE) of DNS-[C6&DC]m were 20 ± 0.4% and 82.13 ± 0.1%, respectively. The BCA assay revealed an optimized membrane-to-DNS ratio of 1:1 (Additional file 1: Fig. S3).

The protein components of the DNS, DNS-C6m, DNS-DCm, and DNS-[C6&DC]m were analyzed by sodium dodecyl sulfate–polyacrylamide gel electrophoresis (SDS-PAGE). The results showed that the original protein compositions of DCm and C6m were retained in DNS-[C6&DC]m (Additional file 1: Fig. S4A). The protein contents of all samples were determined using a BCA kit before western blotting (Additional file 1: Fig. S4B). Compared to the prepared hybrid cell membrane, we found less protein loss due to cell membrane resuspension and ultrasound during the process of preparation. Western blotting analysis for specific protein markers was performed Normally, CD44 and ICAM are related to adhesion and invasion between C6 glioma cells [7, 51, 52]. As shown in Fig. 2K, CD44 and ICAM were expressed on DNS-C6m and DNS-[C6&DC]m, which could generate specific recognition and binding between DNS-[C6&DC]m and glioma cells. In addition, mature DCs mediate T cell activation and proliferation through interactions between ligands (MHC I and CD80) on the DC cell membrane and receptors on the surface of T cells [53]. The MHC I and CD80 protein bands were observed in the DNS-DCm and DNS-[C6&DC]m groups, but not in DNS and DNS-C6m. The results confirmed that the MHC I and CD80 molecules on the nanoparticles retained their T cell binding moieties. Gray value analysis (Additional file 1: Fig. S4C–F) demonstrated that the protein content of DNS-[C6&DC]m was slightly reduced, which could have occurred during the process of preparation. These results further indicated that DNS-[C6&DC]m could potentially serve as a nanoscale NDDS for drug delivery and antigen presentation.

### In vitro release profile

To mimic the release profiles of DTX in vivo, the drug release kinetics of DNS-[C6&DC]m and DNS were measured at 37 °C for 72 h in medium at pH 6.8 to represent the acidic environment of tumor, at pH 7.4 for a normal physiological environment, and at 10% FBS for blood circulation environment [32, 54]. The release results for the different drug formulations are shown in Additional file 1: Fig. S5. DNS showed a similar release behavior in PBS at both pH 7.4 (Additional file 1: Fig. S4A) and pH 6.8 (Additional file 1: Fig. S4B), while an obvious initial burst profile was observed in 10% FBS (Additional file 1: Fig. S4C). Interestingly, DNS-[C6&DC]m only released quickly in PBS at pH 6.8. These results suggest that the stability and controlled release behavior of DNS improves after cell membrane coating, effectively satisfying the drug concentration requirements of chemotherapy. It has been speculated that the mode of entry of the drug into the body can prolong its circulation time.

### Cellular uptake of DNS-[C6&DC]m and the underlying mechanism

**Fig. 3:**
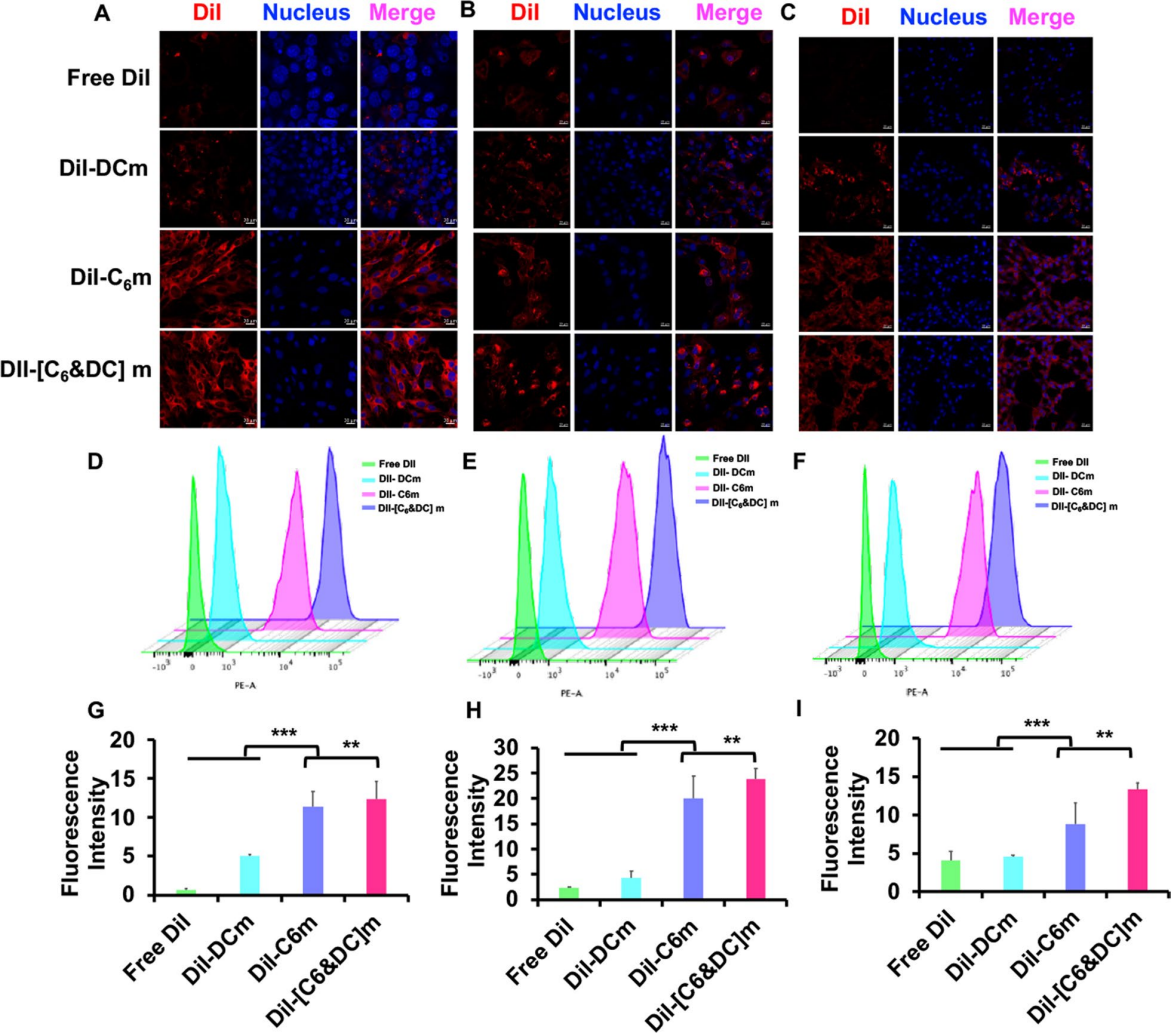
Cell uptake was measured using CLSM and FCM. **A** Confocal fluorescence images of C6 (**A**, **D**, **G**), HUVECs (**B**, **E**, **H**), and bEnd.3 cells (**C**, **F**, **I**) in the presence of Free DiI, DiI-C6m, DiI-DCm, or DiI-[C6&DC]m. The preparations showed different targeting capabilities. (×20 magnification; red: DiI, blue: nuclei; **p < 0.01, ***p < 0.001)

It is well recognized that cellular uptake is a prerequisite for drug delivery. As bEnd.3, HUVECs, and C6 cells are key components of the BBB and BBTB, biomimetic nanosuspension cellular uptake and internalization by these cells were investigated. As shown in Fig. 3, we first studied the targeting and uptake abilities of biomimetic nanosuspensions in C6 glioma cells. Our results demonstrated that endocytosis of different biomimetic nanosuspensions was notably influenced by the type of membrane. The DNS-[C6&DC]m could be taken up by C6 glioma cells at high rates, demonstrating that DNS-[C6&DC]m retains highly specific self-targeting adhesion to C6 glioma cells. To validate the penetrating ability of the biomimetic nanosuspensions into tumor tissues, the cellular uptake of HUVECs, which are similar to tumor neovascular endothelial cells, was studied. DiI-DNS-C6m and DiI-DNS-[C6&DC]m demonstrated stronger intracellular fluorescence in HUVECs compared to free DiI, indicating that C6m can penetrate the BBB and deliver drugs to tumors by targeting tumor neovascularization. Finally, confocal microscopy and flow cytometry demonstrated that the intracellular fluorescence intensity of bEend.3 cells treated with DiI-DNS-DCM was much lower than that of the cells treated with DiI-DNS-[C6&DC]m, indicating that DNS-[C6&DC]m could be better internalized by bEend.3 cells. In addition, the fluorescence intensity of bEend.3 cells in the DNS-C6m group was similar to that in the DNS-[C6&DC]m group, suggesting that the better endocytosis of bEend.3 cells to DNS-[C6&DC]m was the result of C6 membrane-mediated homologous targeting ability.

### In vitro BBB and BBTB model transportation of DNS-[C6&DC]m

**Fig. 4:**
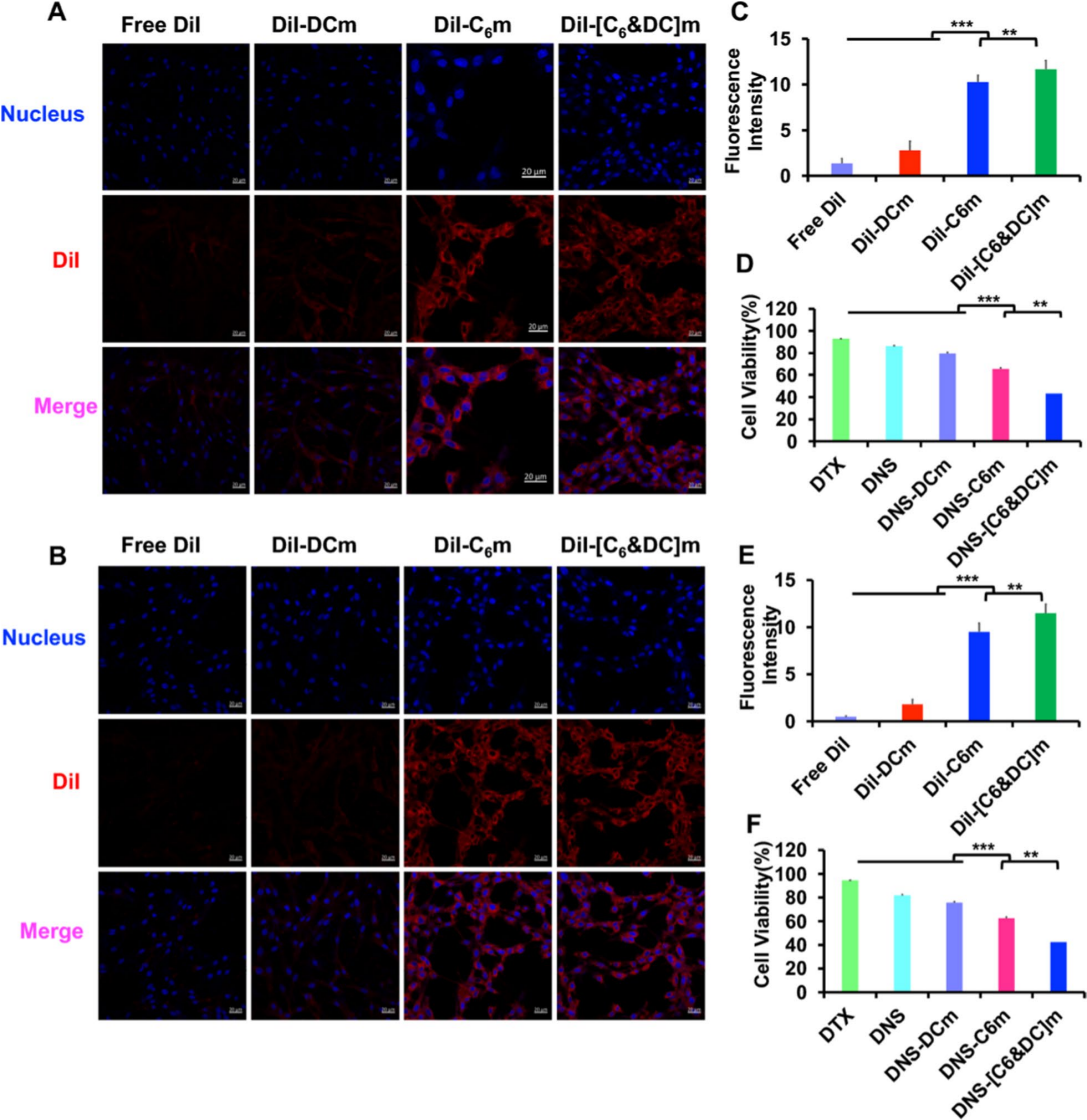
Transcytosis efficiency in the BBB and BBTB models. Cell uptake of different preparations by C6 glioma cells in the bEnd.3/C6 in vitro BBB model (**A**) and in the HUVEC/C6 BBTB model (**B**). The fluorescence intensity of the lower chamber and cell viability in the BBB (**C**, **D**) and BBTB (**E**, **F**) models. (Hoechst 33,258 (blue), DiI (red); ×40 magnification; **p < 0.01, ***p < 0.001)

Transwell models were used to evaluate the ability of DNS-[C6&DC]m to cross the BBB and BBTB in vitro. Monolayer culture in a Transwell with bEnd.3 cells or HUVECs in the upper chamber and C6 cells in the lower chamber are commonly used in in vitro models for brain delivery studies [55]. An in vitro BBB model with barrier function was established using bEnd.3/C6 glioma cells. The TEER value was over 200 Ω cm^2^, indicating that a complete BBB structure was successfully established. Confocal microscopy results revealed that the penetration abilities of DNS-[C6&DC]m and DNS-C6m were stronger than those of the other groups, and the fluorescence intensity of the bottom chamber solution was the highest (Fig. 4A, C). There was a significant difference compared to free DiI and DiI-DNS-DCm, which proved the importance of C6 in crossing the BBB. The HUVEC/C6 cell co-culture model was used as the BBTB, and the resistance value was 321.5 Ω·cm^2^. Among all groups, DNS-[C6&DC]m showed the strongest targeting capability, further indicating that C6m can penetrate the BBB and potentially deliver drugs to tumors (Fig. 4B, E). The apoptosis rates of C6 cells in the BBB and BBTB were determined using the CCK-8 assay. The cell viabilities of DNS-[C6&DC]m, DNS-C6m, DNS-DCm, and DTX in the BBB were 51.13%, 71.67%, 84.21%, and 93.39%, respectively (Fig. 4D), and those in the BBTB were 57.37%, 74.57%, 84.93%, and 92.50%, respectively (Fig. 4F). DNS-[C6&DC]m demonstrated the highest inhibition of cancer cell proliferation. It is reasonable to assume that more DNS-[C6&DC]m could pass through the BBB and BBTB model, reach tumor sites, and lead to better tumor cell killing effects.

### In vitro homologous targeting study

To verify the homotypic targeting effect of DNS-[C6&DC]m, CLSM was used to detect the endocytosis effect of different cells on nanoparticles. The results shown in Additional file 1: Fig. S6 show that the fluorescence intensities of B16, HepG2, and 4T1 cells were much weaker than that of the C6 cells, further demonstrating that DNS-[C6&DC]m has homology targeting and specific recognition functions, which proves that the selective targeting properties of DNS-[C6&DC]m are not suitable for other cancer cells.

### In vitro immune cells priming and tumor cell inhibition

**Fig. 5:**
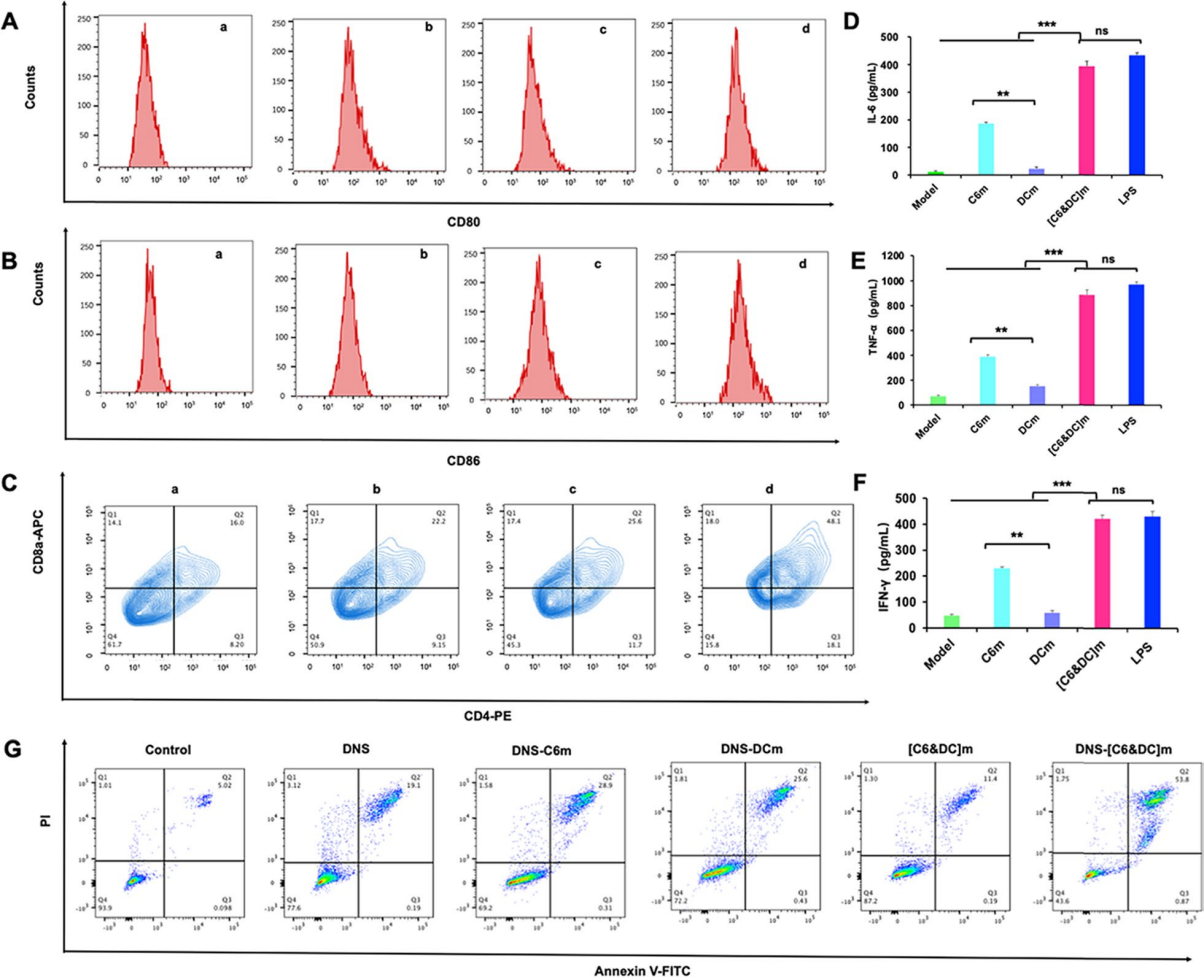
Immune response and cell apoptosis in vitro. **A** Flow cytometric quantification of the expression of CD80 and CD86 (**B**), the markers for DC maturation (**a** PBS; **b** DCm; **c** C6m; **d** [C6&DC]m). **C** Flow cytometric analyses of the expression of CD8a and CD4, the markers for T cells proliferation (**a** PBS; **b** DCm; **c** C6m; **d** [C6&DC]m). **D** The release of IL-6, TNF-α (**E**), and IFN-γ (**F**) by RAW264.7 cells; n = 6. **G** Apoptosis of C6 glioma cells after treatment with different DTX-loaded formulations

The mature of DCs was the key impetus for T cells activation. The hallmarks of DC cell maturation are the high expression of antigen presenting molecules (MHC-I and MHC-II) and stimulating factors (CD80, CD86, etc.) [56, 57]. We assessed the in vitro immunostimulatory activity of DCs after the treatment with C6m, DCm, [C6&DC]m, and the results found that the percentage of CD80^+^ and CD86^+^ DCs after the treatment with [C6&DC]m was obviously higher than that of other groups (Fig. 5A, B and Additional file 1: Fig. S7). Although C6m contained innate tumor antigens, its efficiency of T cell activation seemed to be similar or even lower than that of DMs. This result is possibly related to the specific recognition of DCs by T cells.

Provided that tumor antigens could be processed and expressed on [C6&DC]m during membrane fusion, [C6&DC]m could present tumor antigens to T cells and directly active T cells owing to the partial inclusion of DCm fragments in [C6&DC]m. Like tumor cells, [C6&DC]m can be recognized by DCs and consequently, the matured DCs can serve as APCs to present antigens to T cells. The demonstration of these two of direct and indirect pathways are evaluated via flow cytometry. It is well known that CD8^+^ T lymphocytes (CTLs) and CD4 + helper T cells play a vital role in regulating the immune response [58, 59], we measured the expression of CD8 and CD4 on the cytomembrane of T cells (from mouse splenocytes) via flow cytometry. After 48 h coincubation, the percentage of CD8^+^ CTLs and CD4^+^ helper T cells was dramatically increased (Fig. 5C). In comparison, much less increment was observed in the DNS-C6m and DNS-DCm treated groups. The result indicates that DNS-[C6&DC]m were more powerful to promote a majority of the T cells proliferation.

Macrophages are highly plastic cells that adopt a variety of activation states in response to stimuli that are found in the body environment. In this study, DNS-[C6&DC]m are inherited from two parent cell lines, hybrid membranes could confer a continuous source of tumor antigens, which can be presented for activating macrophages to release cytokines, including IL-6, TNF-α, and IFN-γ [60, 61]. The inflammatory cytokines released by immune cells can help induce the immune system to kill tumor cells, directly or indirectly, thus inhibiting tumor cell growth [62]. To measure the cytokines secreted by RAW264.7 cells after stimulation with [C6&DC]m, the immune-related cytokines, including IL-6, TNF-α, and IFN-γ, were measured using ELISA. After 48 h of coincubation, as shown in Fig. 5D–F, the levels of inflammatory cytokines in the [C6&DC]m group were significantly higher than those in the other groups (*P* < 0.001), which was similar to that of the LPS group. In comparison, a much smaller increment was observed in the C6m- and DCM-treated groups. These results indicate that the hybrid membranes of DCm and C6m were more powerful in stimulating microphages than single C6m or DCm. In the fusion process, DCs can capture and process the tumor antigens of C6s, and then present a whole array of tumor antigens to microphages and further promote the release of TNF-α, IFN-γ, and IL-6 cytokines.

To monitor the effect of released cytokines, the inhibitory effect of cytokines on C6 cells was quantified using the CCK-8 assay. As shown in Additional file 1: Fig. S8, the cell viability of the model group was close to 100%. Compared to the C6m and DCm groups, [C6&DC]m significantly inhibited tumor cell proliferation (*P* < 0.001), indicating that [C6&DC]m could effectively stimulate macrophages to release cytokines (TNF-α, IL-6, and IFN-γ). TNF-α and IFN-γ can bind to their corresponding receptors and then move into the cell. Following uptake by target cell lysosome, cytokines facilitate the reduction of lysosomal stability, resulting in the leakage of various enzymes, which could lead to the lysis of C6 cells. Furthermore, IL-6 can change the glucose metabolism of glioma cells, lower intracellular pH, and cause cancer cell death, further indicating that the fusion of C6m and DCm is necessary for stimulating cells to produce an immune response.

### In vitro cytotoxicity of DNS-[C6&DC]m on C6 glioma cells

As shown in Additional file 1: Fig. S9, DNS-[C6&DC]m effectively inhibited the growth of C6 glioma cells. With increasing drug concentration, the inhibitory effect of each group on C6 cells was found to be significantly enhanced. DNS-[C6&DC]m induced the strongest growth inhibition in C6 cells, with an IC_50_ of 7.78 μg/mL, which was much lower than that of DTX (29.29 μg/mL). The apoptosis analysis via flow cytometry revealed that DNS-[C6&DC]m induced the highest apoptotic rate of 54.26 ± 2.32% (early and late apoptosis) in C6 cells, whereas the rates were 29.52 ± 4.143%, 29.31 ± 2.181%, 19.30 ± 5.021%, and 12.85 ± 2.820% for DNS-C6m, DNS-DCm, DNS, and [C6&DC]m, respectively (Fig. 5G). These results showed that DNS-[C6&DC]m significantly enhanced DTX cytotoxicity and apoptosis in C6 glioma cells, which may be related to the increased cell uptake efficiency and the combination of drug toxicity and immunogenic killing effect, indicating the potential of DNS-[C6&DC]m as an advanced platform for multiple modes of anti-glioma therapy.

### In vivo biodistribution

**Fig. 6:**
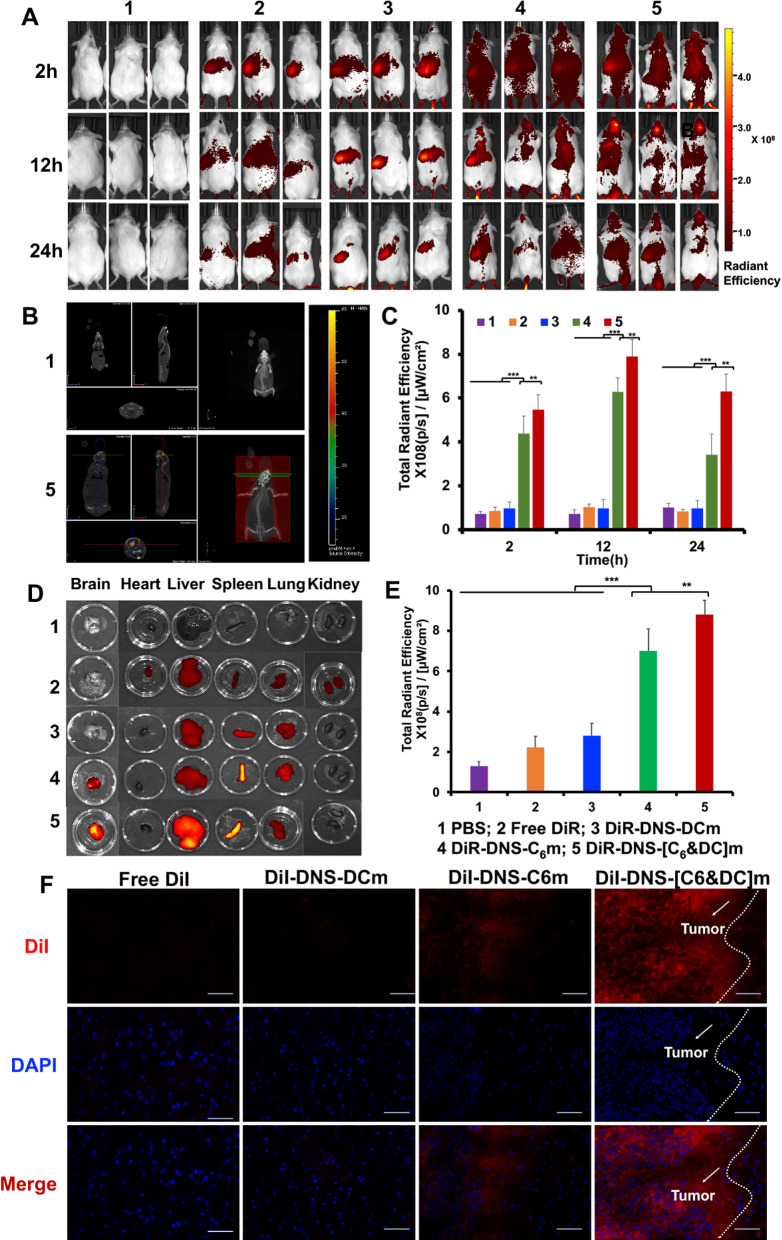
In vivo distribution of DiR-DNS-[C6&DC]m. **A** In vivo fluorescence imaging of intracranial glioma-bearing mice treated with DiR-DNS-[C6&DC]m at different time points. **B** A three-dimensional brain CT tumor localization scan. **C** In vivo quantification of DiR-DNS-[C6&DC]m in brain distribution. **D** Biodistribution of different DiR-labelled biomimetic nanosuspensions across different organs. **E** In vitro quantification of different DiR-labelled biomimetic nanosuspensions in isolated brain. **F** Distribution of brain tissue in vitro (DAPI: blue, DiI: red; 20 × magnification)

The brain tumor targetability of DNS-[C6&DC]m was evaluated in an intracranial glioma-bearing mouse model. We first monitored the in vivo biodistribution of DiR-labeled DTX formulations after intravenous administration in glioma-bearing mice models using an in vivo spectrum imaging system (IVIS® Spectrum, PerkinElmer, USA). As shown in Fig. 6A, compared with the free DiR or DiR-DNS-DCm-treated mice. a strong DiR fluorescence was observed in the DiR-DNS-C6m and DiR-DNS-[C6&DC]m group, localized to brain tumor locations. Notably, DiR-DNS-[C6&DC]m presented a significantly stronger capability of brain targeting in the glioma-bearing mice than any other groups. As the time was extended to 24 h, more DiR-DNS-[C6&DC]m migrated to C6 glioma cells. Furthermore, compared to the free DiR group, DNS-[C6&DC]m demonstrated an increasing trend of the liver accumulation of DNS-[C6&DC]m at first and then a decreasing trend, indicating that the membrane-coated nanomedicine can significantly prolong time of accumulation at the tumor site. Three dimensional CT scan fluorescence imaging results depicted an accumulation of DNS-[C6&DC]m at the tumor site (Fig. 6B), which further supports the precise brain targeting of DNS-[C6&DC]m. These data suggest that the inherent homotypic binding phenomenon among tumor cells resulted in an enhanced brain-tumor-targeting effect of the DiR-DNS-[C6&DC]m, which were able to target the intracranial glioma cells, as further confirmed by a quantitative ROI analysis (Fig. 6C).

We also collected the hearts, livers, spleens, lungs, kidneys, and brains of mice treated with different DiR-labeled DTX formulations and investigated their distribution in each organ (Fig. 6D). Strong DiR-DNS[C6&DC]m and DiR-DNS-C6m fluorescence were observed in the brain and localized to brain tumor locations. Quantitative analysis of the brain tissue further confirmed the brain-targeting effect of DNS-[C6&DC]m (Fig. 6E). Additionally, consistent with previous reports, DiR-DNS[C6&DC]m accumulated in the liver because it is involved in the metabolism and elimination of nanoparticles [42]. Further, the spleen is the largest immune organ, based on the natural antigen presentation ability of DCs, and tumor-associated antigens are presented to the spleen by DNS-[C6&DC]m for immune-activating functions.

The brain tissue of mice injected with DiI-labeled biomimetic nanosuspensions was sectioned. As shown in Fig. 6F, free DiI did not enter the tumor tissues; however, DNS-C6m and DNS-[C6&DC]m were distributed in the tumor tissues, confirming their homologous targeting effect. Taken together, these data confirm our hypothesis that DNS-[C6&DC]m has C6-like tumor-homing characteristics, which facilitate homotypic-driven DNS-[C6&DC]m recruitment at the glioma site.

### In vivo immune activation effect

**Fig. 7:**
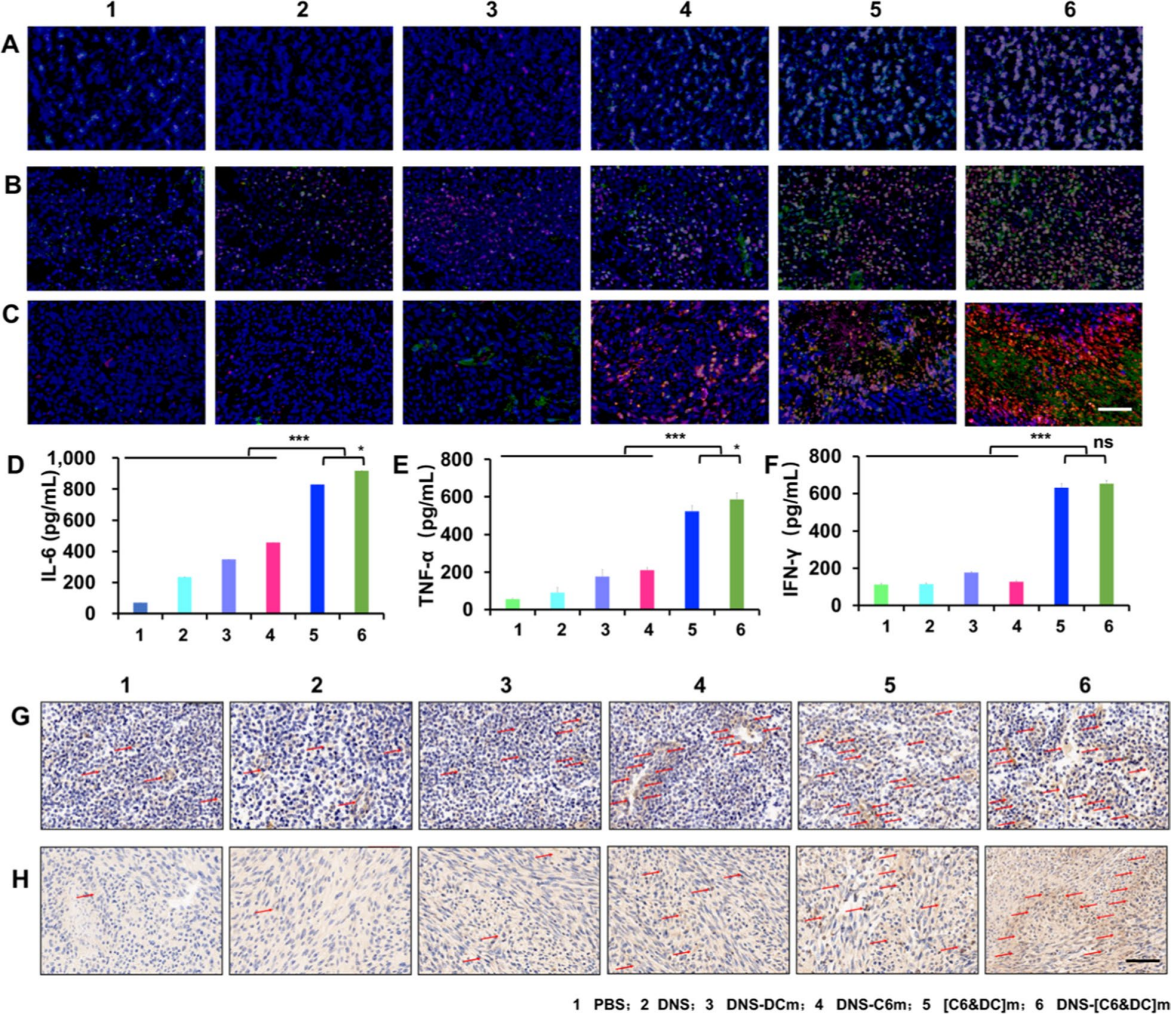
Immune stimulation efficiency in different tissues and mice serum. **A** Immunofluorescence staining of CD8 (Red) and CD4 (Green) antibodies in spleen and lymph node (**B**). **C** Immunofluorescence staining of DiI (Red), CD8 (Green) and CD4 (pink) antibodies in brain of glioma-bearing mice. **D** Release of cytokines IL-6, TNF-α (**E**), and IFN-γ (**F**) in mice serum. **G** Immunohistochemical staining of TNF-α of the lymph node and tumors (**H**) (**p < 0.01, ***p < 0.001, ns, not significant; n = 6; ×40 magnification)

Next, we evaluated the antitumor immune responses elicited by the injection of DNS-[C6&DC]m in vivo. CD8^+^ CTLs are the main force that kills cancer cells in our immune design, and CD4^+^ T cells can help CTLs proliferate and increase their toxicity towards tumors [63, 64]. We measured the expression of CD8 and CD4 at tumor sites with different treatments via IHC and immunofluorescence staining to investigate the direct immune response pathway. As shown in Fig. 7A, compared to the PBS group, the number of CD8^+^ and CD4^+^ T cells from the spleen in the [C6&DC]m and DNS-[C6&DC]m groups increased significantly. In comparison, a much smaller increase was observed in the DNS-C6m- and DNS-DCm-treated groups. Although DNS-C6m contains tumor antigens from C6m, its efficacy in T cell activation seemed to be similar or even lower than that of DNS, which fully proves that DNS-[C6&DC]m relies on the antigen presentation effect of DCm to induce an immune response from the body. In the fusion process, DNS-[C6&DC]m can retain the tumor antigens of tumor cells, and mature DCs stimulated by tumor-associated antigens can promote the proliferation and activation of CD8^+^ and CD4^+^ T lymphocytes. Remarkably, a similar phenomenon was observed in the lymph nodes (Fig. 7B). Furthermore, it is important to find accurate colocalization in the glioma site of the biodistribution of DiI-labeled DNS-[C6&DC]m and cytotoxic T cells (Fig. 7C), further indicating that DNS-[C6&DC]m can not only accurately cross the BBB and target the glioma site for drug delivery directly, but can also effectively stimulate the immune system to produce cytotoxic T cells for indirect immunogenicity inhibition of the blood circulation on the tumor [65].

Cytokine secretion is a characteristic marker of T cell activation. Inspired by the activation of microphages in vitro, the release of TNF-α, IFN-γ, and IL-6 in mouse serum was examined using ELISA after different treatments. As shown in Fig. 7D–F, the expression of these cytokines in the DNS-[C6&DC]m group was higher than that in the other groups. Notably, despite the fact that DNS-[C6&DC]m and DNS-C6m both contain tumor-associated antigens, the levels of these three cytokines were significantly different (*P* < 0.001), which proved that DNS-[C6&DC]m relies on the antigen presentation effect of DCm to induce an immune response. The delivery of antigens to macrophages affects the microenvironment of the immune system, which may potentially lead to immunogenic cell damage. IHC was used to observe TNF-α expression within the lymph nodes and tumor sites (Fig. 7G, H). Upregulation of TNF-α expression was observed after DNS-[C6&DC]m treatment, which was consistent with the in vitro results, indicating that the DNS-[C6&DC]m treatment promoted macrophage activity and induced comparable TNF-α production. TNF-α binds to its corresponding receptors and then moves into the cell. Following uptake by the target cancer cell lysosome, the cytokine reduces lysosomal stability, leading to the lysis of C6 cells.

In summary, the in vivo immune experimental results demonstrated that DNS-[C6&DC]m could effectively strengthen the immune response and inhibit the immune escape of tumor cells. Further, DNS-[C6&DC]m could stimulate microphages to release cytokines, which bind to the corresponding receptors and then move into the cell. Following uptake by the target cell lysosome, cytokines reduce lysosomal stability, leading to the leakage of various enzymes and C6 cell lysis. Additionally, DNS-[C6&DC]m could enhance the ability of T lymphocytes to capture antigens. Naive T cells differentiate into CD8^+^ and CD4^+^ T cells and indirectly interfere with tumor growth [66].

### In vivo anti-glioma efficacy

**Fig. 8:**
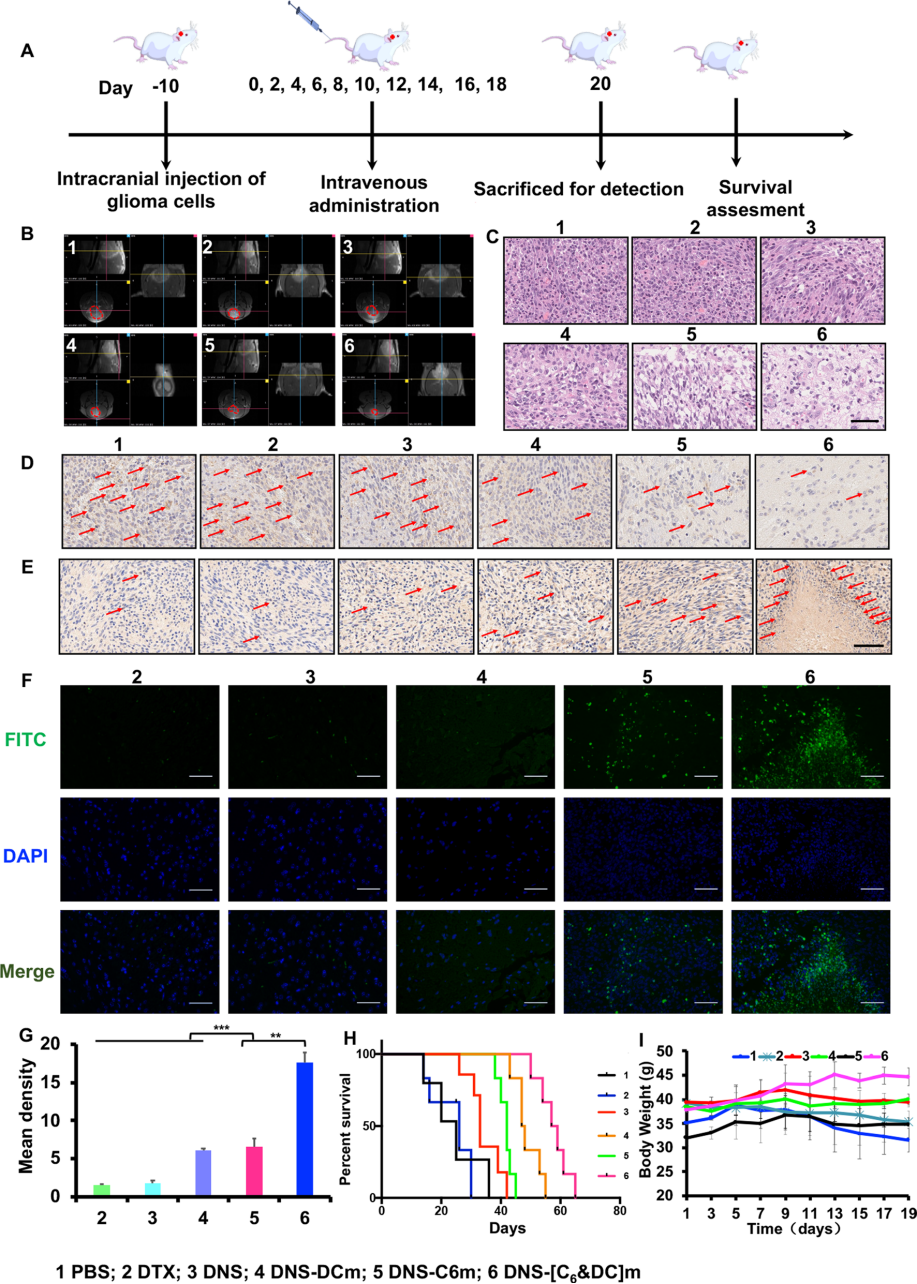
Therapeutic efficacy of DNS-[C6&DC]m in glioma-bearing mice. **A** Schematic illustration of the administration regimenfor DNS-[C6&DC]m therapy. **B** MRI of normal and glioma-bearing mice after different treatments. **C** H&E staining of the tumor tissues. **D** Immunohistochemical staining of CD31 and Caspase-3 (**E**) of the tumors. **F** TUNEL staining analysis of the tumors (×40 magnification). **G** Quantification using ImageJ of TUNEL apopotosis after different administration (**p < 0.01, ***p < 0.001, ns, not significant). **H** Kaplan–Meier survival curve of glioma-bearing mice after drug treatment. **I** Body weight changes of mice in different treatment groups

To explore the therapeutic efficacy of DNS-[C6&DC]m in vivo, we monitored the contours of the different glioma sites in C6 glioma-bearing mice after DNS-[C6&DC]m, DNS-C6m, DNS-DCm, DNS, free DTX, and PBS treatment using MRI. As shown in Fig. 8B, glioma cells in the PBS group grew rapidly and exhibited the largest tumors. For the free DTX and DNS groups, a malignant situation similar to that of the PBS group was observed, and the glioma proliferated significantly with time. In contrast, DNS-[C6&DC]m and DNS-C6m groups showed weak MRI signals, and glioma growth was obviously suppressed, indicating that these formulations could suppress the rapid growth of the glioma more effectively through accurate delivery of DTX to the brain tumor site as a result of the homotypic targeting abilities of C6m. High drug-carrying capacity significantly improved the drug concentration at the targeted sites. Notably, better antitumor efficacy was observed in the DNS-[C6&DC]m group than in the DNS-C6m group, possibly due to the immune response induced by DCm. The combination of drug delivery and antigen delivery resulted in a better chemoimmunotherapeutic effect in gliomas.

Further, we performed histological analysis to examine the therapeutic effects of the different treatments. H&E staining revealed typical glioma malignancy with dense glioma cell features in both the PBS, DTX, DNS, and DNS-DCm groups. In comparison, glioma cell density was significantly lower in DNS-[C6&DC]m-treated mice than in the mice of the other groups, demonstrating that DNS-[C6&DC]m effectively inhibited tumor proliferation (Fig. 8C). CD31 is a marker of tumor angiogenesis [67]. IHC staining of brain tissue was conducted to examine the expression of CD31 receptors in neovascularization related to tumor cell proliferation. The relatively high brain tumor CD31 expression after DNS-[C6&DC]m treatment highlights DNS-[C6&DC]m suppression of malignant progression in glioma (Fig. 8D).

Apoptosis of brain tumor tissue was investigated by performing caspase-3 staining and TUNEL staining (Fig. 8E–G). Cell apoptosis was more evident in the DNS-[C6&DC]m group than in any other group, indicating that DNS-[C6&DC]m could penetrate more deeply into the glioma tissues via drug delivery, and the activation of the immune system for immunotherapy killed more viable cells than any other treatment. Kaplan–Meier survival curves (Fig. 8H) demonstrated that treatment with DNS-[C6&DC]m significantly prolonged the survival duration of glioma-bearing mice. The survival duration of the mice treated with DNS-[C6&DC]m (65 days) was significantly longer than that of the mice treated with PBS (36 days), free DTX (37 days), DNS (42 days), DNS-DCm (45 days), and DNS-C6m (55 days). In terms of physiological status, decreased body weight was observed in PBS- and DTX-treated mice, while a relatively lower rate of body weight decrease was observed in mice receiving DNS-[C6&DC]m treatment (Fig. 8I). In DTX-treated mice, this decreased body weight may have been a result of systemic side effects of DTX resulting from non-specific circulation of chemotherapeutic drug in the blood [68]. This phenomenon is consistent with DNS-[C6&DC]m enhancing the intracranial glioma targetability and therapeutic efficacy of DTX, consequently ameliorating DTX side effects.

### Safety of biomimetic drug-delivery systems in vitro and in vivo

**Fig. 9:**
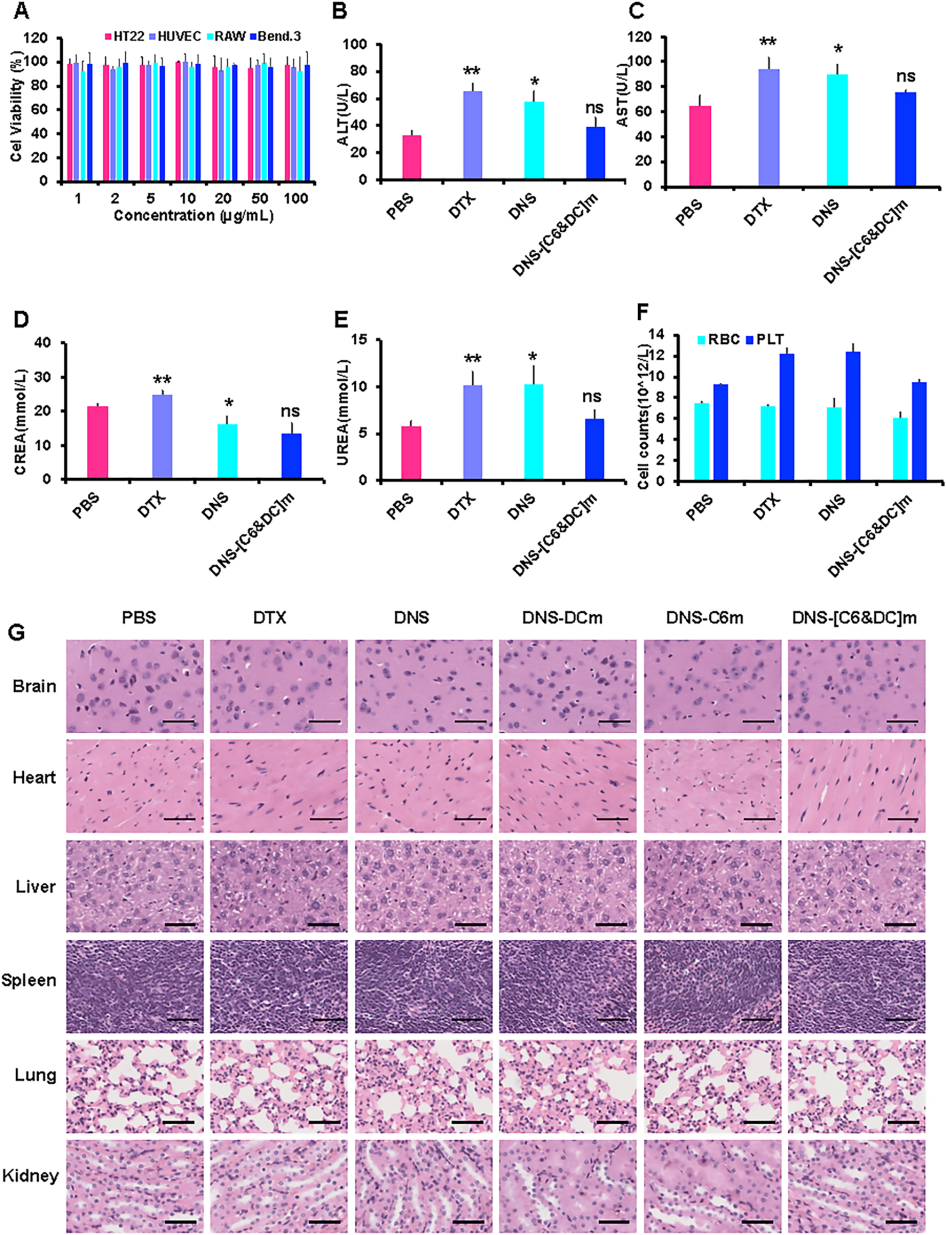
Preliminary safety evaluation. **A** Cell viabilities of HT22 cells, HUVECs, RAW cells, and bEnd.3 cells incubated for 24 h with different concentrations of empty formulations. Data are presented as means ± SD (n = 6). **B** Levels of liver and kidney function markers, including UREA, AST (**C**), ALT (**D**), and CREA (**E**). **F** Levels of blood cells, including DC and platelets. **G** Histological examination of major organs derived from mice after treatment (×40 magnification). (**P < 0.01, *P < 0.05, ns, not significant)

An ideal nanocarrier needs to not only have suitable physical and chemical properties but also minimal toxicity and high biocompatibility. The in vivo biosafety of the biomimetic nanosuspensions was evaluated using hematological and histopathological analyses. Evaluation of uric acid, aspartate aminotransferase, alanine aminotransferase, and creatinine levels in the blood showed that DTX and DNS affect liver and kidney function. As shown in Fig. 9B–E, the mice treated with DNS-C6m, DNS-DCm, and DNS-[C6&DC]m were relatively stable. We also measured the levels of platelets and RBCs in the blood. Compared to the indices of the mice in DTX and DNS groups, the indices of the DNS-C6m-, DNS-DCm-, and DNS-[C6&DC]m-treated mice were found to be relatively stable. In contrast, mice treated with the free drugs DTX and DNS had increased levels of platelets and RBCs during the treatment period (Fig. 9F). Additionally, after the administration of DTX, DNS, DNS-C6m, DNS-DCm, and DNS-[C6&DC]m, no obvious pathological damage to the heart, liver, spleen, lung, kidney, and brain of the mice was observed (Fig. 9G). The histological and blood biochemistry results confirmed the lower toxicity and better in vivo safety of DNS-[C6&DC]m, possibly due to the enhanced stability, minimized RES uptake, and controlled drug release profile.

## Conclusions

In summary, we have successfully established a hybrid cell membrane-coated drug nanosuspension platform for multiple modes of anticancer therapy via drug and antigen delivery. As nature-inspired biomimetic nanosuspensions, DNS-[C6&DC]m are expected to maintain the excellent biological functions of both C6 and DC cells. Our observations suggest that the homologous targeting of biological characteristics of cancer cell membranes provides a key impetus for precise targeted delivery of drugs, which increases drug accumulation and further enhances the efficacy of the loaded chemotherapeutic drug in tumor sites of the intracranial glioma model. At the same time, due to the professional antigen presentation characteristic of DCm, the platform presents antigens to the body's immune system for efficient downstream immune activation. The synergistic effects of chemotherapy and immunotherapy increase the anticancer effect. For clinical translation, we predict that the hybrid membrane coating technology presented in this study can be further expanded to other cell types for different biomedical applications. By fusing the homologous cancer membrane with immune-related cells, diverse therapeutic nanoparticles can be freely camouflaged according to the disease and drug needs to achieve efficient drug delivery and strengthen immune response. Further, this platform may be generalized for the delivery of various drugs with low solubility or high toxicity, especially those that need to be administered intravenously, such as hydroxycamptothecin and paclitaxel. Overall, our research demonstrates the outstanding therapeutic efficiency of DNS-[C6&DC]m for glioma treatment, which has the potential to treat a wide range of cancers through precise tumor-targeted drug delivery and antigen presentation, and represents a promising approach to individualized multiple-mode therapeutics for cancer therapy.

## Supplementary Information

**Additional file 1: Fig. S1.**Characterization of DNS. (**A**) X-ray diffraction patterns of different components in the DNS. (**B**) Scanning of different components in the DNS using differential scanning calorimetry. (**C**) FTIR spectra of different components in DNS. **Fig. S2.** Zeta potential of different preparations, as measured using the DLS. **Fig. S3.** Quantification of total proteins on DNS-[C6&DC]m by BCA assay after incubating different amount of [C6&DC]m to DNS at different membrane-to-DNS weight ratios (w/w). **Fig. S4.** (**A**) The membrane protein of the biomimetic nanosuspensions was determined via SDS-PAGE. (**B**) The protein contents of cancer cell membranes and biomimetic nanosuspensions were determined using the BCA kit. (**C**) Gray value of membrane-specific proteins ICAM, CD44 (**D**), MHC I(**E**), and CD80 (**F**). **Fig. S5** Release profile of free DTX, DNS, and DNS-[C6&DC]m in (**A**) PBS at pH 7.4, (**B**) PBS at pH 6.8, or (**C**)10% FBS at 37 °C. Error bars: mean ± SD (n = 3). **Fig. S6** Cell uptake of the biomimetic nanosuspensions by different cancer cells was investigated using CLSM. The intracellular uptake of DNS-[C6&DC]m in B16 (**A**), HepG2 (**B**), 4T1 (**C**), and C6 glioma (**D**) cells. The nuclei were stained with Hoechst 33258 (blue), and the DNS-[C6&DC]m were labeled with DiI (red) (40× magnification). **Fig. S7.** The percentage of DCs maturation. (*p <0.05, **p <0.01, ***p <0.001, ns, not significant; n = 6). **Fig. S8.** The inhibitory effect of cytokines on C6 glioma cells. (*p <0.05, **p <0.01, ***p <0.001, ns, not significant; n= 6). **Fig. S9.** The inhibitory effect of different DTX-loaded formulations on C6 glioma cells. Error bars: mean ± SD (n = 6).
