# Supplementary material for: Hybrid membrane-coated nanosuspensions for multi-modal anti-glioma therapy via drug and antigen delivery
Source: J Nanobiotechnology. 2021 Nov 20;19:378. doi: 10.1186/s12951-021-01110-0 (PMC8606100; doi:10.1186/s12951-021-01110-0)
Supplement: Supplementary file 1 — Additional file 1: Fig. S1.Characterization of DNS. (A) X-ray diffraction patterns of different components in the DNS. (B) Scanning of different components in the DNS using differential scanning calorimetry. (C) FTIR spectra of different components in DNS. Fig. S2. Zeta potential of different preparations, as measured using the DLS. Fig. S3. Quantification of total proteins on DNS-[C6&DC]m by BCA assay after incubating different amount of [C6&DC]m to DNS at different membrane-to-DNS weight ratios (w/w). Fig. S4. (A) The membrane protein of the biomimetic nanosuspensions was determined via SDS-PAGE. (B) The protein contents of cancer cell membranes and biomimetic nanosuspensions were determined using the BCA kit. (C) Gray value of membrane-specific proteins ICAM, CD44 (D), MHC I(E), and CD80 (F). Fig. S5 Release profile of free DTX, DNS, and DNS-[C6&DC]m in (A) PBS at pH 7.4, (B) PBS at pH 6.8, or (C)10% FBS at 37 °C. Error bars: mean ± SD (n = 3). Fig. S6 Cell uptake of the biomimetic nanosuspensions by different cancer cells was investigated using CLSM. The intracellular uptake of DNS-[C6&DC]m in B16 (A), HepG2 (B), 4T1 (C), and C6 glioma (D) cells. The nuclei were stained with Hoechst 33258 (blue), and the DNS-[C6&DC]m were labeled with DiI (red) (40× magnification). Fig. S7. The percentage of DCs maturation. (*p <0.05, **p <0.01, ***p <0.001, ns, not significant; n = 6). Fig. S8. The inhibitory effect of cytokines on C6 glioma cells. (*p <0.05, **p <0.01, ***p <0.001, ns, not significant; n= 6). Fig. S9. The inhibitory effect of different DTX-loaded formulations on C6 glioma cells. Error bars: mean ± SD (n = 6). [file 12951_2021_1110_MOESM1_ESM.docx]

**Additional information**

**
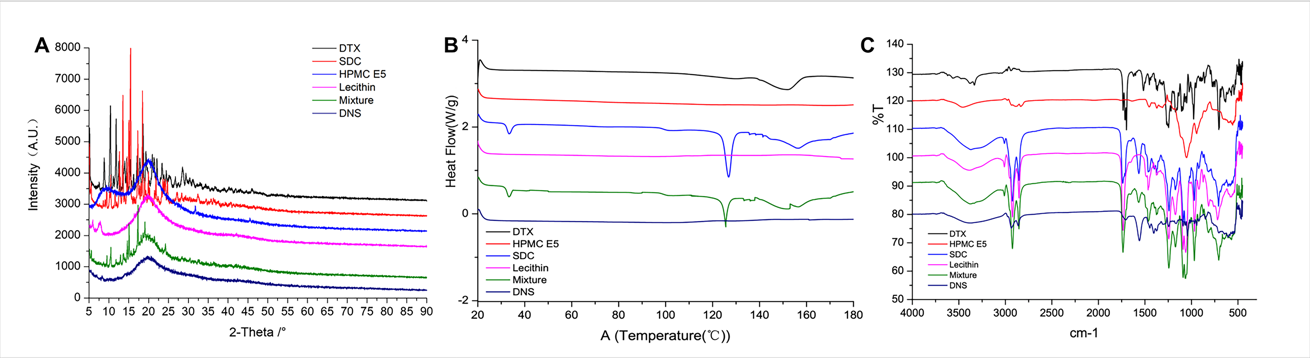
**

**Fig. S1** Characterization of DNS. (A) X-ray diffraction patterns of different components in the DNS. (B) Scanning of different components in the DNS using differential scanning calorimetry. (C) FTIR spectra of different components in DNS.


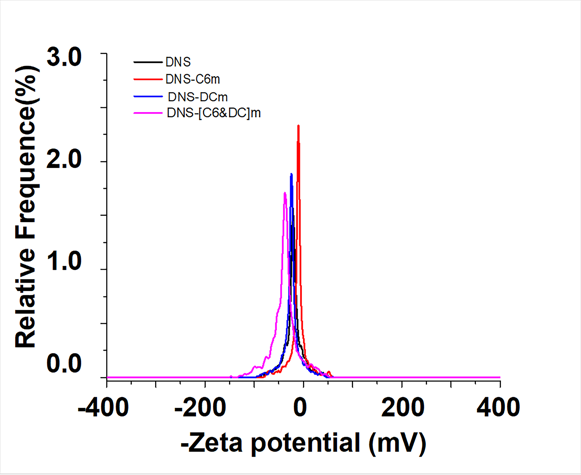


**Fig. S2** Zeta potential of different preparations, as measured using the DLS.


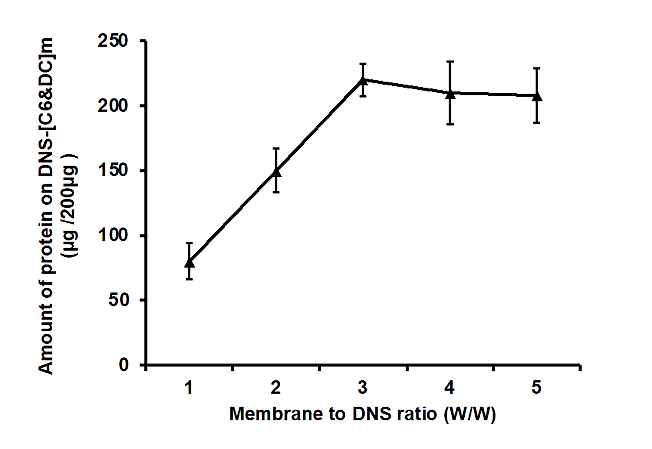


**Fig. S3** Quantification of total proteins on DNS-[C6&DC]m by BCA assay after incubating different amount of [C6&DC]m to DNS at different membrane-to-DNS weight ratios (w/w).

**
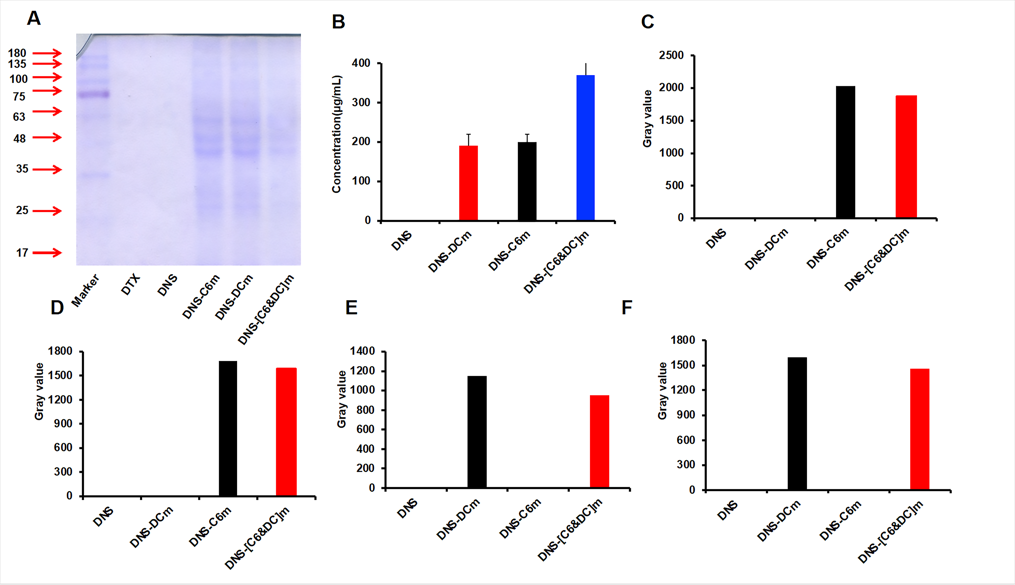
**

**Fig. S4** (A) The membrane protein of the biomimetic nanosuspensions was determined via SDS-PAGE. (B) The protein contents of cancer cell membranes and biomimetic nanosuspensions were determined using the BCA kit. (C) Gray value of membrane-specific proteins ICAM, CD44 (D), MHC I(E), and CD80 (F).


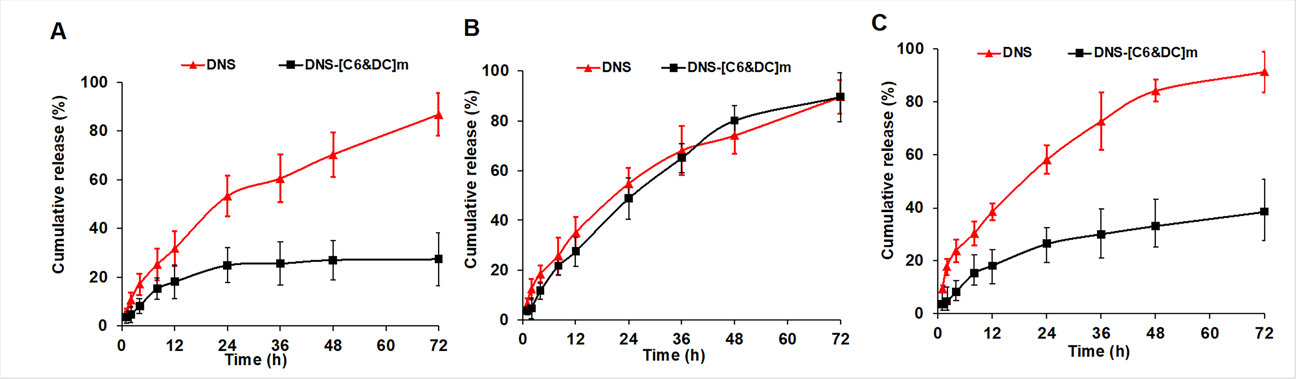


**Fig. S5** Release profile of free DTX, DNS, and DNS-[C6&DC]m in (A) PBS at pH 7.4, (B) PBS at pH 6.8, or (C) 10% FBS at 37 °C. Error bars: mean ± SD (n = 3).


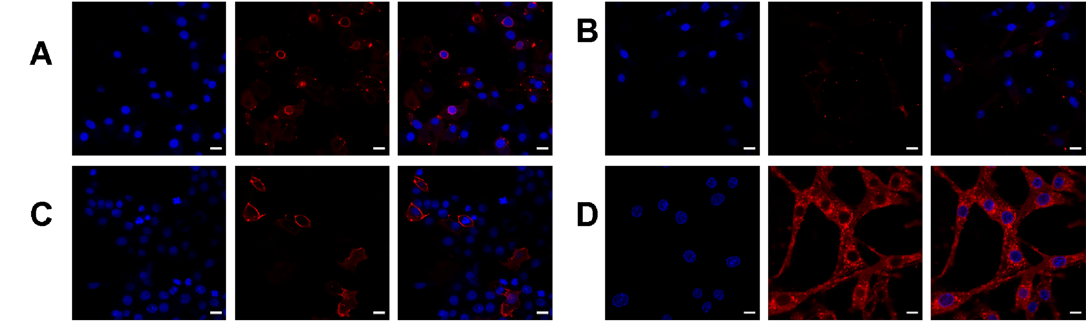


**Fig. S6** Cell uptake of the biomimetic nanosuspensions by different cancer cells was investigated using CLSM. The intracellular uptake of DNS-[C6&DC]m in B16 (A), HepG2 (B), 4T1 (C), and C6 glioma (D) cells. The nuclei were stained with Hoechst 33258 (blue), and the DNS-[C6&DC]m were labeled with DiI (red) (40× magnification).

**Fig. S7** The percentage of DCs maturation. (**p* <0.05, ***p* <0.01, ****p* <0.001, ns, not significant; n = 6)


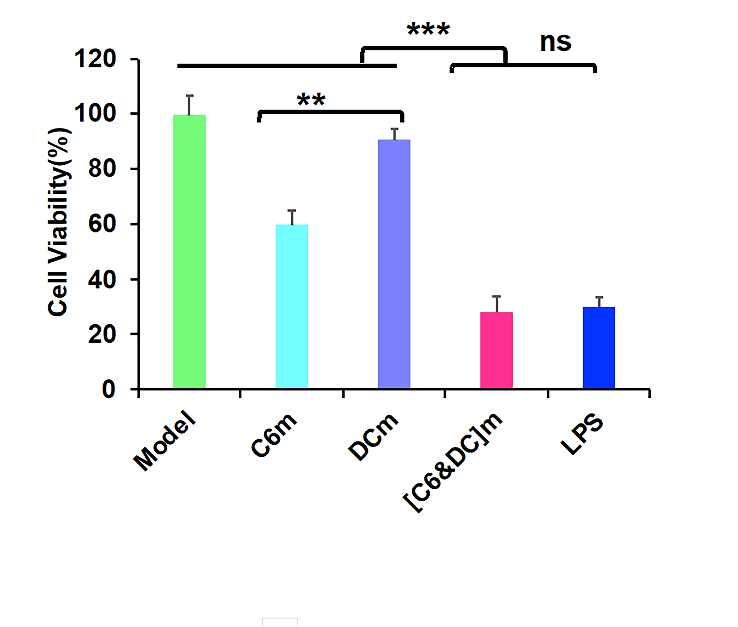


**Fig. S8** The inhibitory effect of cytokines on C6 glioma cells. (*p <0.05, **p <0.01, ***p <0.001, ns, not significant; n = 6).

**
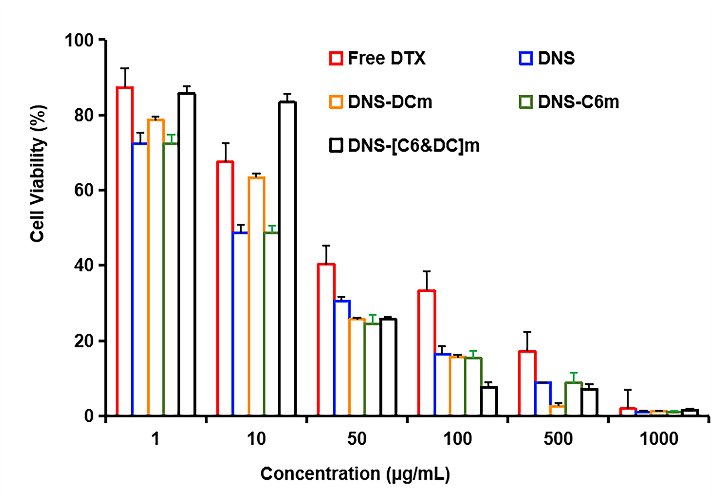
**

**Fig. S9** The inhibitory effect of different DTX-loaded formulations on C6 glioma cells. Error bars: mean ± SD (n = 6).
